# Human metapneumovirus Induces Reorganization of the Actin Cytoskeleton for Direct Cell-to-Cell Spread

**DOI:** 10.1371/journal.ppat.1005922

**Published:** 2016-09-28

**Authors:** Farah El Najjar, Nicolás Cifuentes-Muñoz, Jing Chen, Haining Zhu, Ursula J. Buchholz, Carole L. Moncman, Rebecca Ellis Dutch

**Affiliations:** 1 Department of Molecular and Cellular Biochemistry, University of Kentucky, Lexington, Kentucky, United States of America; 2 Laboratory of Infectious Diseases, National Institute of Allergy and Infectious Diseases, Bethesda, Maryland, United States of America; National Institutes of Health, UNITED STATES

## Abstract

Paramyxovirus spread generally involves assembly of individual viral particles which then infect target cells. We show that infection of human bronchial airway cells with human metapneumovirus (HMPV), a recently identified paramyxovirus which causes significant respiratory disease, results in formation of intercellular extensions and extensive networks of branched cell-associated filaments. Formation of these structures is dependent on actin, but not microtubule, polymerization. Interestingly, using a co-culture assay we show that conditions which block regular infection by HMPV particles, including addition of neutralizing antibodies or removal of cell surface heparan sulfate, did not prevent viral spread from infected to new target cells. In contrast, inhibition of actin polymerization or alterations to Rho GTPase signaling pathways significantly decreased cell-to-cell spread. Furthermore, viral proteins and viral RNA were detected in intercellular extensions, suggesting direct transfer of viral genetic material to new target cells. While roles for paramyxovirus matrix and fusion proteins in membrane deformation have been previously demonstrated, we show that the HMPV phosphoprotein extensively co-localized with actin and induced formation of cellular extensions when transiently expressed, supporting a new model in which a paramyxovirus phosphoprotein is a key player in assembly and spread. Our results reveal a novel mechanism for HMPV direct cell-to-cell spread and provide insights into dissemination of respiratory viruses.

## Author Summary

Human metapneumovirus (HMPV) is an important human respiratory pathogen that affects all age groups worldwide. There are currently no vaccines or treatments available for HMPV, and key aspects of its life cycle remain unknown. We examined the late events of the HMPV infection cycle in human bronchial epithelial cells. Our data demonstrate that HMPV infection leads to formation of unique structures, including intercellular extensions connecting cells, and large networks of branched cell-associated filaments. Viral modulation of the cellular cytoskeleton and cellular signaling pathways are important for formation of these structures. Our results are consistent with the intercellular extensions playing a role in direct spread of virus from cell-to-cell, potentially by transfer of virus genetic material without particle formation. We also show that the HMPV phosphoprotein localizes with actin and can promote membrane deformations, suggesting a novel role in viral assembly or spread for paramyxovirus phosphoproteins.

## Introduction

Human metapneumovirus (HMPV) is a major cause of acute upper and lower respiratory tract infections worldwide [1–6]. It was originally identified in 2001 in patients with symptoms similar to human respiratory syncytial virus (HRSV) infection [7], but studies have shown that HMPV has been circulating in human populations for more than 50 years [8,9]. Between 5–20% of hospitalizations due to respiratory infections in young children are caused by HMPV [10,11]. It is also a significant cause of morbidity and mortality in immunocompromised and elderly populations [12,13], and a recent report indicated that hospitalization rates of older adults infected with HMPV are similar to those of influenza infections [14]. Clinical presentation of infection can range from cough, fever, rhinitis and wheezing to more severe symptoms including bronchiolitis, croup, asthma exacerbation, and pneumonia. Currently, there are no specific antiviral treatments or vaccines for HMPV infections, and the major form of treatment is supportive therapy [15,16].

HMPV is a member of the family *Paramyxoviridae*, genus *Pneumovirinae*, which includes enveloped viruses with a negative sense, single stranded RNA genome. HMPV particles, similar to other paramyxoviruses, are highly pleomorphic in shape with both spherical and filamentous morphologies reported [5,7,13]. The HMPV genome is approximately 13,000 nucleotides in length and encodes for three surface glycoproteins: the fusion protein (F), the attachment protein (G), and the small hydrophobic protein (SH), all of which are densely packed on the viral envelope. The genome also encodes a matrix protein (M), and five proteins that are associated with the RNA genome: nucleocapsid protein (N), phosphoprotein (P), large polymerase protein (L), and the M2-1 and M2-2 proteins. Studies over the past decade have resulted in a better understanding of the mechanisms of entry and fusion of HMPV [17–19]. However, the late stages in the replication cycle during which viral components assemble and exit the cell are not well understood. The HMPV M protein, similar to other paramyxovirus M proteins, plays an essential role in production of virus particles, and budding of HMPV has been shown to occur in an endosomal sorting complex required for transport (ESCRT) independent manner [20]. Formation of HMPV virus like particles (VLPs) occurs following co-expression of the F and M proteins, with the G protein enhancing this process, thus indicating an important role for these proteins in the HMPV assembly process [18,21]. In addition, morphogenesis of HMPV occurs at lipid-raft microdomains that are rich in actin, suggesting a role for lipid rafts and the actin cytoskeleton in virus assembly [22].

A substantial body of evidence indicates that spread of some enveloped viruses, including hepatitis C virus, rabies virus and several members of the herpesvirus, retrovirus, and poxvirus families, can occur directly from cell-to-cell without diffusion through the extracellular environment [23–33]. The mechanisms by which these viruses subvert cellular processes for cell-to-cell spread can vary substantially; however, manipulation of the cell cytoskeleton is often involved [34–38]. For paramyxoviruses, cell-to-cell spread independent of particle release occurs for measles virus across neuronal synapses and fusion pores in respiratory epithelial cells and for HRSV through syncytia formation in cultured cells [39–43]. Recently, it was reported that influenza A virus and the paramyxovirus parainfluenza virus 5 (PIV5) can also spread directly between cells in a neutralizing antibody independent manner [44].

In this study, we sought to characterize the late steps of HMPV infection in human bronchial epithelial cells and to identify host factors critical to this stage of the replication cycle. We demonstrate that HMPV infection results in remodeling of the cell cytoskeleton leading to the formation of extensive branched networks of cell-associated virus filaments and stimulation of intercellular extensions where the major viral structural proteins and viral RNA localize. Our results indicate that actin dynamics are critical for the formation of these structures since treatment of cells with actin depolymerizing or stabilizing drugs or targeted inhibition of the major regulators of actin dynamics, the Rho GTPases Rac1, Cdc42 and RhoA, decreased the formation of branched viral filamentous networks and intercellular extensions. Interestingly, transient expression of the HMPV P protein resulted in formation of membrane extensions similar to those seen in late stages of HMPV infection, suggesting a novel role for this protein in HMPV egress. A proximity ligation assay revealed, for the first time, co-localization between a paramyxovirus P protein and actin. Studies of HMPV spread in a co-culture assay indicated a novel mode of direct cell-to-cell spread for HMPV which, in contrast to cell-free infection, can occur in the presence of neutralizing antibodies and does not require the attachment factor heparan sulfate. Analysis of the involvement of the cell cytoskeleton in direct cell-to-cell spread in the co-culture assay showed a major role for the actin cytoskeleton, Cdc42, and Rac1 in HMPV spread, thus supporting the association of intercellular extensions with enhanced intercellular spread, and providing new insights into the pathogenesis of this important human respiratory pathogen.

## Materials and Methods

### Cell lines

BEAS-2B cells, obtained from ATCC, were grown in Bronchial Epithelial Cell Growth Medium (BEGM) containing all the recommended supplements (Lonza). 16HBE cells [45], kindly provided by Dieter C. Gruenert, University of California, San Francisco, were grown in Minimum Essential Medium with Earle’s salt (Invitrogen) plus 10% fetal bovine serum (FBS) and 2mM L-glutamine (Invitrogen). A549 cells (provided by Hsin-Hsiung Tai, University of Kentucky) were maintained in Roswell Park Memorial Institute medium (RPMI; Lonza) supplemented with 10% FBS. COS-7 cells and LLC-MK2 cells, both obtained from American Type Culture Collection (ATCC), and Vero cells, kindly provided by Robert Lamb, HHMI/Northwestern University, were grown in Dulbecco's modified Eagle's medium (DMEM; Gibco) supplemented with 10% FBS. CHO-K1 and pgsA745 cells [46], obtained from ATCC, and pgsD677 [47], provided by Jeff Esko, University of California, San Diego, CA, were grown in HyClone Ham's F-12, Kaighn's modification medium (Thermo Scientific, Waltham, MA) supplemented with 10% FBS.

### Plasmids, antibodies and reagents

The coding region for CAN 97–83 HMPV F was subcloned into the pCAGGS mammalian expression plasmid (kindly provided by Jun-ichi Miyazaki, Osaka University Medical School) as previously described [48]. A codon optimized HMPV N gene was cloned into pCAGGS. The coding sequence for HMPV M was synthesized in a pUC57 vector (Genetech, Arcade, NY) and subcloned into pCAGGS. Full length cDNA for HMPV P was amplified by RT-PCR using RNA isolated from HMPV CAN97-83 and cloned into pCAGGS. Antibodies for HMPV N protein (ab94801), P protein (ab94803) and F protein (ab94800) were obtained from Abcam. A polyclonal antibody against the avian metapneumovirus C M protein, kindly provided by Sagar M. Goyal (University of Minnesota, Minneapolis, MN), which has been shown to cross-react with HMPV M, was used to detect HMPV M protein [20]. Filamentous actin was detected using phalloidin, and the antibody for tubulin was purchased from the Proteintech Group (66031). Secondary antibodies conjugated with fluorescein isothiocyanate (FITC), tetramethylrhodamine (TRITC), Atto488 or Alexa flour 647 were obtained from Jackson ImmunoResearch. AlexaFluor 647 conjugated wheat germ agglutinin (Invitrogen) was used for plasma membrane staining. Drugs for disruption of the actin cytoskeleton or microtubules were purchased as indicated: cytochalasin D (C8273; Sigma), latrunculin A (428021; Calbiochem), jasplakinolide (sc-202191; Santa Cruz), paclitaxel (580556; Calbiochem), nocodazole (487928; Calbiochem), rhosin (555460; Calbiochem), NSC 23766 (sc-204823; Santa Cruz), ML 141 (4266; Tocris).

### Virus propagation and titer determination

HMPV and recombinant, green fluorescent protein (GFP)-expressing HMPV (rgHMPV) strain CAN97-83 (genotype group A2), were propagated in Vero cells or LLC-MK2 as previously described [17]. Titers of rgHMPV were determined by performing serial dilutions of the virus samples on a 96-well plate, incubating for 24 hours followed by counting the number of GFP positive cells. For determination of titers of non-GFP expressing HMPV, virus samples were subjected to serial dilution, 100 μl of virus was added to LLC-MK2 cells and incubated at 37°C for 1 hour, followed by overlay with Opti-MEM containing 0.75% methylcellulose. After 4–5 days, cells were fixed in 10% formalin, incubated with anti-HMPV F antibody followed by a peroxidase-conjugated secondary antibody (Kirkegaard and Perry Labs, KPL). TrueBlue peroxidase substrate (KPL) was added and incubated for 10 minutes at room temperature, and plaques were then counted. To determine cell-associated virus titers, infected cells were washed three times with PBS, then scraped in Opti-MEM containing 1x sucrose phosphoglutamate followed by three freeze-thaw cycles. For determination of titers of released virus, cell culture media was collected, centrifuged at 2,500 × rpm for 15 min at 4°C on a Sorvall RT7 tabletop centrifuge followed by centrifugation on a 20% sucrose cushion at 135,000 x g and 4°C for 150 minutes using a SW41 swinging bucket rotor on a Beckman ultracentrifuge. Recombinant GFP-expressing PIV5 (rgPIV5) [49], provided by Robert Lamb (Howard Hughes Medical Institute, Northwestern University) was propagated in MDBK cells and titered as previously described [50].

### HMPV purification by sucrose density gradient centrifugation for mass spectrometry analysis

BEAS-2B cells were mock infected or infected with HMPV, and virus propagation was performed as described above. Cell culture media was collected and centrifuged at 2500 x rpm for 15 minutes on a Sorvall RT7 tabletop centrifuge. The supernatant was then subjected to centrifugation on a 20% sucrose cushion for 150 minutes at 135,000 x g at 4°C using a SW28 swinging bucket rotor on a Beckman Optima L90-K ultracentrifuge. Virus pellets were then resuspended in 1x Tris-sodium-EDTA (TNE) buffer, layered onto a 30%-45%-60% (weight/volume) discontinuous sucrose gradient and centrifuged at 150,000 x g for 90 minutes at 4°C. The band containing the virus at the 30%-45% interface was collected and centrifuged on a 30% to 60% continuous sucrose gradient for 18 hours at 135,000 x g and 4°C. The virus-containing band was then pelleted on a 20% sucrose cushion. The pellet was resuspended in 1x TNE buffer and centrifuged at 400 x g for 10 minutes using an Amicon Ultra Filter Unit to get rid of low molecular weight contaminants. The purified virus was then subjected to mass spectrometric analysis.

### Mass spectrometry for detection of HMPV-associated cellular proteins

The virus solution was denatured with 8M urea and subjected to dithiothreitol reduction and iodoacetamide alkylation. The sample was then diluted to a 2M final concentration of urea and digested in-solution with trypsin. The tryptic peptides were subjected to shot-gun proteomics analysis as previously described [51]. Briefly, LC-MS/MS analysis was performed using an LTQ-Orbitrap mass spectrometer (Thermo Fisher Scientific, Waltham, MA) coupled with an Eksigent Nanoflex cHiPLC system (Eksigent, Dublin, CA) through a nano-electrospray ionization source. The peptide samples were separated with a reversed phase cHiPLC column (75 μm x 150 mm) at a flow rate of 300 nL/min. For identifying host proteins in purified HMPV particles, the LC-MS/MS data were submitted to a local mascot server for MS/MS protein identification via Proteome Discoverer (version 1.3, Thermo Fisher Scientific, Waltham, MA) against the *Homo sapiens* (human) taxonomy subset of Swissprot database. Typical parameters used in the MASCOT MS/MS ion search were: trypsin digest with maximum of two miscleavages, cysteine carbamidomethylation, methionine oxidation, a maximum of 10 ppm MS error tolerance, and a maximum of 0.8 Da MS/MS error tolerance. A decoy database was built and searched. Filter settings that determine false discovery rates (FDR) were used to distribute the confidence indicators for the peptide matches. Peptide matches that pass the filter associated with the strict FDR (with target setting of 0.01) were assigned as high confidence.

### Immunofluorescence and confocal microscopy

Cells grown on 10 mm coverslips were infected with HMPV or PIV5, and at various times post infection, cells were washed in phosphate buffered saline (PBS) and fixed in 4% paraformaldehyde (PFA) for 15 minutes at room temperature. Cells were then permeabilized in 1% Triton X-100 for 15 minutes at 4°C followed by blocking in 1% normal goat serum (NGS) and incubated with the corresponding primary antibody overnight at 4°C. The following day, cells were washed with 0.05% tween-PBS, secondary antibodies were added, and cells were incubated at 4°C for one hour. Coverslips were then mounted on glass slides using Vectashield mounting media containing 4',6-diamidino-2-phenylindole (DAPI) for staining the nucleus (Vectorlabs, Burlingame, CA). Pictures were taken using a Nikon 1A confocal microscope and analyzed with the NIS-Elements software. All images were processed in Adobe Photoshop, with equivalent adjustments made to all panels.

### Immunostaining for Stochastic Optical Reconstruction Microscopy (STORM)

BEAS-2B cells grown in glass bottom 35mm dishes were fixed in 3% PFA for 15 minutes followed by reduction in 0.1% sodium borohydride (NaBH_4_) for 7 minutes at room temperature. Cells were then washed three times with PBS, five minutes per wash while shaking, and permeabilized in 0.2% Triton X-100 for 15 minutes prior to blocking in 10% NGS/0.05% Triton for 90 minutes at room temperature. Primary antibodies diluted in 5% NGS/0.05% Triton were then added and incubated for 60 minutes followed by washing five times in 1%NGS/0.05% Triton and incubating with the secondary antibody for 30 minutes. Washes using 1%NGS/0.05% Triton were then performed five times followed by post fixation in 3% PFA for 10 minutes, three washes with PBS and two washes with distilled water. Cells were stored at 4°C until imaging using a Nikon Super Resolution Microscope N-STORM, and image processing was performed using NIS-elements software.

### Virus spread in a methyl cellulose overlay

BEAS-2B cells were inoculated with rgHMPV or rgPIV5 at an M.O.I. of 2. Six hours post infection, infection media was removed, cells were washed two times with PBS, and Opti-MEM containing 0.75% methylcellulose was then added. Images were taken every 24 hours using a 5x objective of a Zeiss Axiovert 100 microscope.

### Co-culture assay for direct cell-to-cell-spread of HMPV

BEAS-2B cells were infected with rgHMPV at an M.O.I. of 2 for 48 hours and then incubated with 7μM cell tracker orange CMRA (Life Technologies, # C34551) for 30 minutes at 37°C. Cells were then washed 5 times with PBS to remove any bound virus particles, lifted with trypsin and added to uninfected BEAS-2B target cells at a ratio of 1:1. Neutralizing antibodies, DS7 (10 μg/ml) and 54G10 (0.4 μg/ml), were then added and cells incubated for an additional 24 hours. Afterwards, cells were collected, fixed in 1% formaldehyde and analyzed by flow cytometry. Direct cell-to-cell spread was defined as the percentage of GFP-only positive cells normalized to percentage of double positive (GFP/cell tracker CMRA orange) donor cells. To test the role of heparan sulfate in direct cell-to-cell spread of HMPV, BEAS-2B donor cells were infected and stained with cell tracker orange CMRA as mentioned above and incubated with CHO, pgsD677 or pgsA745 cells at a ratio of 1:1 and co-cultured for 24 hours.

#### Flow cytometry analysis

For determination of percentage of target cells infected in the coculture assay, cells were fixed in 1% formaldehyde diluted in PBS with 50mM EDTA, and analyzed with a BD FACSCalibur (BD, Franklin Lakes, NJ). Expression of GFP and cell tracker CMRA orange of at least 50,000 cells was determined, and data analysis was performed using BDFACS software (BD Biosciences). The percentage of GFP-expressing cells was normalized to percentage of double positive donor cells in each condition followed by normalization to control samples.

### Stellaris Fluorescent *in situ* hybridization (FISH) for viral RNA detection

Forty-eight DNA probes targeting the HMPV vRNA genome between nt 1–5467 were obtained from BioSearch Technologies (Novato, CA) and designed using the software provided by the company. Each probe is 20 nt long and linked at the 3’end to Quasar 570 fluorophore. BEAS-2B cells grown in 8-well chamber slides were infected with rgHMPV at an MOI of 1. Twenty-four, 48 and 72 h.p.i., cells were fixed for 10 min with 4% PFA and then permeabilized overnight with 70% ethanol at 4°C. The next day cells were washed once with 2x SSC-10% formamide buffer, and then incubated overnight at 25°C in hybridization buffer (4x SSC, 1x Denhardt’s solution, 150 μg/mL ssDNA, 2mM EDTA, 10% Dextran Sulphate in DEPC treated water) containing the probes at a concentration of 2.5 mM. After 24 hrs, cells were washed two times for 20 min with 2x SSC-10% formamide buffer, and slides were then mounted using Vectashield mounting media.

### Proximity ligation assay (PLA)

BEAS-2B cells grown on 10mm coverslips were mock infected or infected with HMPV at an M.O.I. of 2, and 24 h.p.i., cells were fixed with 4% PFA and permeabilized with 1% Triton X-100-PBS. Cells were then incubated in blocking solution at 37°C for 2 hours. A mouse primary antibody for HMPV P and a rabbit β-actin antibody or rabbit α tubulin antibody were added and incubated overnight at 4°C. A proximity ligation assay was then performed using Duolink In Situ red mouse/rabbit kit (Sigma, DUO92101). PLA probes diluted 1:5 were added, and cells were incubated for 1 hour at 37°C in a humidified chamber and processed for ligation for an additional 30 minutes at 37°C. Cells were then washed twice, polymerase was added and DNA was amplified with a florescent substrate for 100 min at 37°C. Coverslips were then mounted on glass slides using Vectashield, and images were taken on a Nikon A1 confocal laser microscope. Images were processed and analyzed for total fluorescent intensity using BlobFinder software.

### Sholl analysis

Cells were prepared for immunofluorescence as described above and images were acquired using a Nikon A1 confocal microscope. A total of 10 cells per condition from three separate experiments were manually traced and the total length of intercellular extensions was determined using NeuronJ plug-in for NIH ImageJ analysis tool. To quantitate the degree of branching, Sholl analysis was performed using the Sholl analysis plug-in for ImageJ. Briefly, a series of concentric circles of 10 μm radii intervals were drawn around the center of the cell body, and the number of intersections at each circle was determined.

### Statistical analysis

Statistical analysis was performed using Prism6 for Windows software (Graphpad). A *p* value ≤ 0.05 was considered statistically significant. For all graphs, mean values ± standard deviation (SD) are shown.

## Results

### HMPV buds primarily as cell-associated filamentous networks

**Fig 1:**
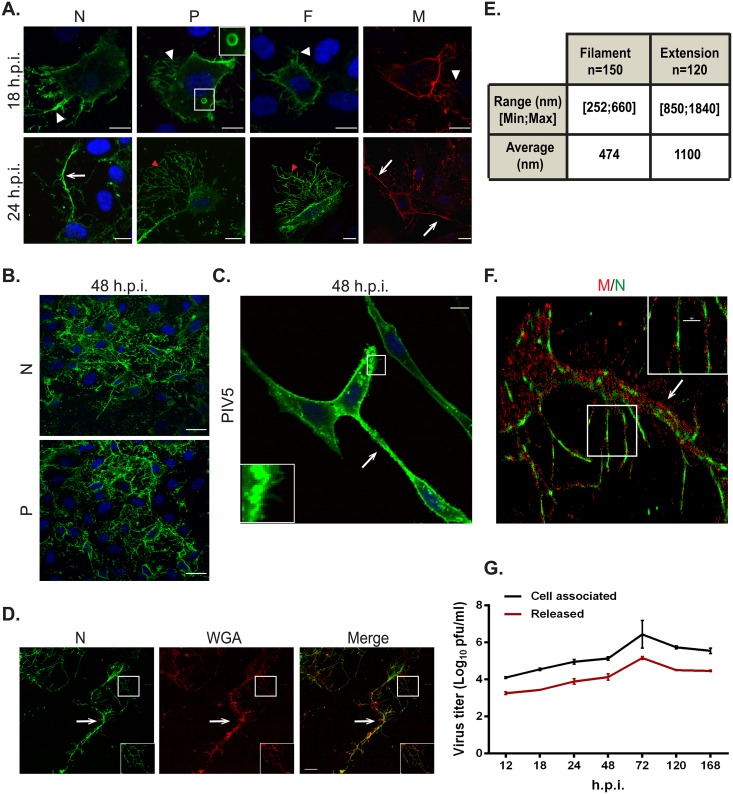
HMPV infection in BEAS-2B cells results in the formation of branched filamentous networks and intercellular extensions. (A) HMPV infected BEAS-2B cells at 18 or 24 h.p.i., were fixed and processed for immunofluorescence staining. White arrows indicate intercellular extensions, red arrowheads indicate branched viral filaments and white arrowheads indicate viral filaments. Scale bars = 10 μm. (B) HMPV infected BEAS-2B cells at 48 h.p.i. were fixed, processed for immunofluorescence and stained with antibodies for HMPV N or P. Scale bar = 50 μm. (C) BEAS-2B cells were inoculated with PIV5 and at 48 h.p.i. cells were fixed, processed for immunofluorescence and stained with antibody against the F protein of PIV5. Arrow indicates an intercellular extension in a PIV5-infected cell and inset shows filaments. Scale bar = 10 μm. (D) BEAS-2B cells were inoculated with HMPV at M.O.I. of 3, and at 24 h.p.i; cells were fixed and stained with the plasma membrane marker WGA and immunostained for HMPV N. Scale bar = 10 μm. (E) Table showing range and average diameter of filaments and intercellular extensions. Images were taken for a total of 100 HMPV-infected cells and imageJ analysis tool was used to determine the diameter of branched filaments and intercellular extensions. (F) Cells were inoculated with HMPV, and 24h later, cells were processed for Stochastic Optical Reconstruction Microscopy (STORM) and stained with an anti-N antibody (green) and an anti-M antibody (red). Inset shows filaments with a central core of N protein surrounded by matrix protein. Scale bar = 1 μm. (G) BEAS-2B cells were inoculated with rgHMPV at an M.O.I. of 2, and at different times post infection, cells or culture media were collected and virus titers determined. Graph shows mean ± SD for three independent experiments.

Production of paramyxovirus particles is generally a multistep process that occurs in the cytoplasm of an infected cell and culminates in the assembly of virus components at the plasma membrane followed by budding and release of infectious virus particles [52,53]. HMPV lacking the envelope proteins G and SH has been shown to be infectious *in vitro* and *in vivo* [54,55], indicating that assembly of infectious HMPV particles does not require G and SH proteins. To investigate the late stages of the HMPV replication cycle, we determined the cellular localization of the main viral structural proteins, the N and P proteins, which are bound to the viral genome and are part of the ribonucleoprotein (RNP) complex, the internal protein M, and the envelope F protein, in infected human bronchial epithelial cells, BEAS-2B. By 18 h.p.i., viral proteins were located primarily at the plasma membrane and in filaments protruding from the plasma membrane (white arrowheads, Fig 1A). In addition, the P protein (Fig 1A, 18 h.p.i., inset) and N protein were seen in discrete cytoplasmic structures or inclusion bodies. Inclusion body formation has been associated with infection for a number of negative sense RNA viruses. The precise role of inclusion bodies in paramyxovirus infection is not well understood; however, some studies indicate the presence of RNA genomes in these bodies suggesting that these are sites of active RNA replication [56–58]. Several respiratory viruses, including influenza virus, RSV and PIV3 form filaments at the plasma membrane [59–61], and it was recently shown that HMPV buds as filamentous structures in LLC-MK2 cells [21]. Interestingly however, by 24 h.p.i., viral proteins were seen primarily in cell-associated branched filaments (Fig 1A, red arrowhead) that formed extensive filamentous networks as infection progressed to 48 h.p.i. (Fig 1B). Viral proteins also localized in thicker and longer structures that ran between infected cells, which we termed intercellular extensions (Fig 1A, white arrows), though the open or closed nature of these extensions could not be determined from this analysis. These extensions were seen extending from opposite sides of a single cell, indicating that they are not retraction fibers (Fig 1A, lower panel right). In the majority of cases, extensions connected two cells, though some extensions were observed that emerged from one cell without connecting to a second cell. Filaments containing viral proteins were also seen projecting from these intercellular extensions. PIV5 has been previously reported to form intercellular extensions [44], but the number and extent of extensions was much greater in HMPV-infected BEAS-2B cells compared to PIV5-infected BEAS-2B cells, and extensive branching filament structures were not observed from PIV5 infection, though individual budding filaments were evident (Fig 1C). Staining with the plasma membrane marker WGA showed that both intercellular extensions (arrow) and filaments (inset) from HMPV infected cells are extensions of the plasma membrane (Fig 1D). To clearly differentiate between filaments and intercellular extensions, we measured the diameter of these structures using the ImageJ analysis tool. Intercellular extensions were thicker, ranging from 850 nm to 1840 nm, with an average diameter of 1100 nm compared to 470 nm for the filaments with a range of 252 nm to 660 nm (Fig 1E).

To further examine the structure of the filaments and intercellular extensions, super-resolution microscopy was performed using a STORM imaging system. Cellular extensions (Fig 1F, arrow) were seen protruding from the cell body (left side of image) with M and N localized throughout the length of the extensions. M and N also localized in branched filaments protruding from the cell body and the cellular extensions. An organized localization of M and N was seen within the filaments, with the N protein observed in the core of the filament surrounded by the M protein (Fig 1F, inset). The organized localization of M and N in the branched filaments revealed by STORM, and the similar morphology to tomographic images of HRSV [62], suggest that these structures are associated with filamentous budding of HMPV. Determination of HMPV titers at different hours post-infection showed that titers of cell-associated virus were 0.5–1 log higher than those of released virus particles throughout the infection period in BEAS-2B cells, indicating that HMPV is primarily cell associated (Fig 1G). Previous studies of HMPV infection in LLC-MK2 cells indicated that the virus was mainly cell-associated and that budding particles were filamentous [22,63]. However, the extensive branched networks and intercellular extensions visualized during HMPV infection in a more physiologically relevant model of human bronchial epithelial cells have not been previously reported for paramyxoviruses.

### Cell-associated branched HMPV filaments are actin based

**Fig 2:**
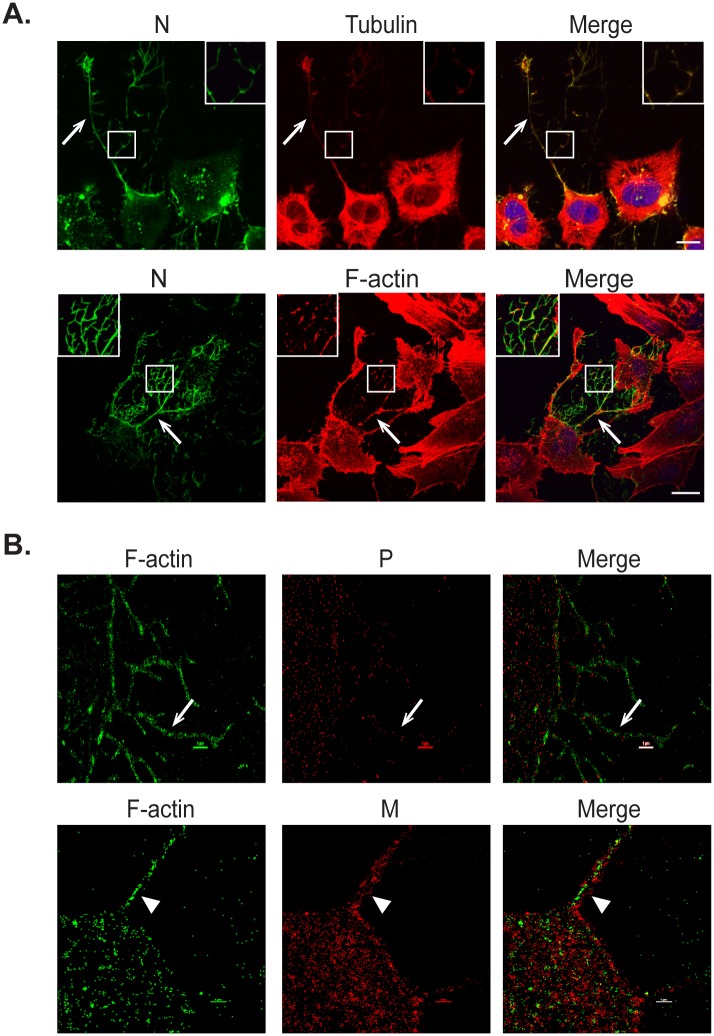
Actin and tubulin are present in intercellular extensions and branched filamentous networks. (A) BEAS-2B cells were inoculated with HMPV, and 24 h.p.i. cells were fixed and stained for HMPV N, tubulin or F-actin. Inset shows colocalization of actin and tubulin with N in branched filaments and arrows indicate intercellular extensions. Scale bar = 50μm. (B) Cells were infected with HMPV for 24h, processed for Stochastic Optical Reconstruction Microscopy (STORM) and stained with an anti-P or anti-M antibody (red) and phalloidin (green). Arrows show a branched filament and arrowheads show a filament. Scale bar = 1 μM.

Plasma membrane dynamics and shape are regulated by the cell cortex, composed primarily of F-actin, with microtubules involved through regulation of the structure of cortical F-actin [64]. To determine the contribution of the cell cytoskeleton to the formation of both the cell-associated branched filaments and intercellular extensions, infected BEAS-2B cells were co-stained for viral proteins and F-actin or tubulin. Both tubulin and F-actin co-localized with HMPV N in budding viral filamentous networks (Fig 2A, inset). Tubulin and F-actin were also present in intercellular extensions (Fig 2A, arrows). The staining for N and F-actin was more intense in the intercellular extensions and branched filaments than in the cell body, while that of tubulin was faint in these structures. In addition, high resolution microscopy showed localization of actin (green) in the branched filaments (Fig 2B arrow) and in the inner core of a filament (Fig 2B arrowhead) budding from the cell body along with viral proteins M and P (red) respectively indicating the close association of F-actin with the budding structures in HMPV-infected cells. This is in contrast to what has been reported for RSV since F-actin was excluded from the viral filaments in RSV infected cells [65], but actin has been reported in purified measles virus preparations [66].

**Fig 3:**
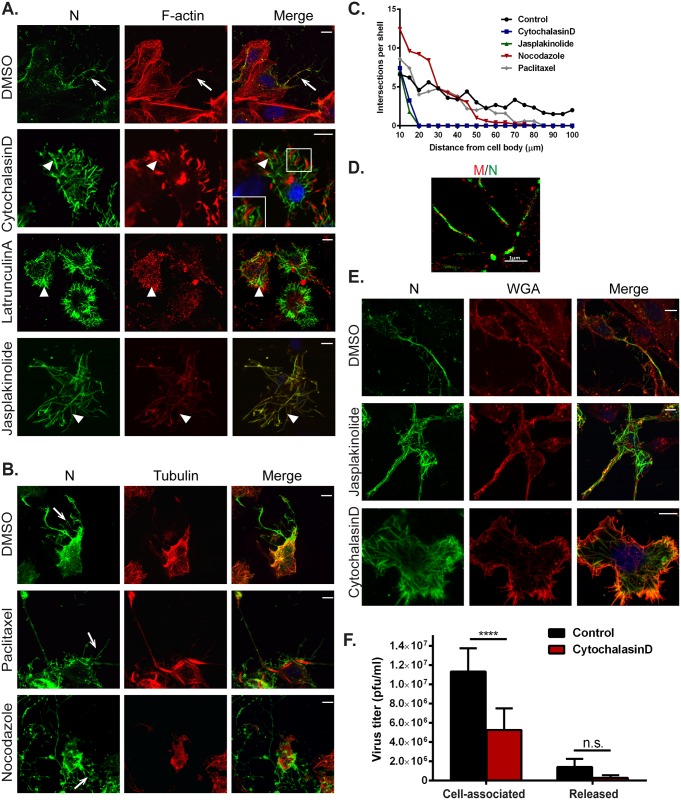
Actin and microtubules have different roles in formation of branched filamentous networks and intercellular extensions. (A) and (B) BEAS-2B cells, were inoculated with HMPV and 2 h.p.i. DMSO vehicle or the indicated drug was added, and cells were incubated for an additional 22 hours. Cells were then fixed and processed for immunofluorescence staining. Arrows indicate branched filamentous networks and arrowheads indicate viral filaments. Scale bars = 10 μm. (C) Cells treated as in A and B were processed for immunofluorescence and images acquired. The NeuronJ plugin of the ImageJ analysis tool was used to manually trace 10 cells for each condition from three independent experiments. Sholl analysis was then performed to evaluate level of branching. (D) Cells were inoculated with HMPV for 2 hours and treated with cytochalasin D. Twenty-four h.p.i. cells were processed for STORM and stained with an anti-N antibody (green) and an anti-M antibody (red). (E) BEAS-2B cells were infected with HMPV, drugs were added at 2 h.p.i. and at 24 h.p.i, cells were fixed and stained with WGA and antibody for N. Scale bars = 10μm. (F) Cells were inoculated with rgHMPV at an M.O.I. of 1 and treated with inhibitors at 2 h.p.i. At 48 h.p.i., cells or culture media were collected and virus titers determined. Graph shows mean ± SD for three independent experiments. Statistical analysis was performed using two-way analysis of variance (ANOVA), (*** = *p*< 0.0001; n.s. = non-significant).

To further investigate if the formation of HMPV filamentous networks was dependent on actin and/or microtubules, cells were infected for two hours and then treated with either DMSO or with inhibitors of actin or microtubule dynamics until fixation at 24 h.p.i. Disruption of actin polymerization using cytochalasin D (2 μM) or latrunculin A (500nM) or treatment of cells with jasplakinolide (150 nM) to stabilize actin filaments resulted in loss of the extensive filamentous HMPV networks seen in DMSO control treated cells (Fig 3A, arrow), indicating an important role of active actin dynamics in the budding of the complex filamentous networks (Fig 3A). However, intracellular filaments containing HMPV N were seen in cells treated with cytochalasin D, latrunculin A and jasplakinolide (Fig 3A, arrowheads), and actin was seen in close proximity to the filaments (Fig 3A, cytochalasin D, inset), suggesting that budding and elongation, but not initial filament formation by viral proteins, requires actin dynamics. In contrast, disruption or stabilization of microtubule polymerization using nocodazole (17 μM) or paclitaxel (12.5 μM), respectively, did not prevent the formation of branching HMPV filamentous networks indicating that microtubule dynamics are not required for budding of cell-associated filamentous HMPV (Fig 3B, arrows). To obtain a quantitative measurement of the effect of the different inhibitors on the formation of branched filaments, we performed Sholl analysis on infected cells. This method of image quantitation is commonly used to measure the complexity of dendritic branching in neurons by creating concentric circles around the cell body and determining the number of intersections at a defined distance from the center of the cell body [67]. Consistent with the microscopic images, disruption of microtubules did not affect the extent of branched filaments, whereas inhibition of actin dynamics prevented formation of the branched filamentous networks (Fig 3C). STORM imaging showed intracellular filaments containing N and M in cells treated with cytochalasin D, and the localization of N and M in these filaments resembled that of control DMSO treated cells (Fig 1E) with the N protein on the inside and M on the outside (Fig 3D). WGA staining showed deformation of the plasma membrane coinciding with viral protein-containing filaments, suggesting that these filaments can partially deform the plasma membrane (Fig 3E). These observations indicate that assembly of HMPV proteins into initial filamentous structures present within the infected cell is driven primarily by viral proteins and possibly other host factors but does not require actin polymerization. However, actin polymerization is required for efficient budding of HMPV filamentous networks from the plasma membrane. To further test the role of the actin cytoskeleton in HMPV particle production, we determined the effect of actin disruption by cytochalasinD on titers of HMPV particles. Cells were infected with HMPV and 2 hours later, DMSO control or cytochalasin D were added and 48 h.p.i., cells and culture media were collected to determine titers. Inhibition of actin polymerization resulted in a statistically significant decrease of greater than two-fold in titers of cell associated HMPV (Fig 3F), indicating a role for an intact actin cytoskeleton in production of infectious cell-associated particles. A reduction was also observed for released particles, though it did not rise to the level of statistical significance.

A role of actin in the HMPV infection cycle was further supported by the detection of a large amount of actin in purified HMPV particles by mass spectrometry analysis (S1 Table). To confirm that the cellular proteins detected in the purified HMPV particles were specific and were not contaminants from released exosomes, we performed the same purification steps and mass spectrometry analysis on media collected from uninfected cells. As seen in S2 Table, keratin was the most abundantly detected cellular protein in supernatants collected from mock infected cells, whereas actin was identified with only 2 peptides. In contrast, actin was detected in high amounts in purified HMPV virions with more than 20 peptides (S1 Table). In addition, several other actin associated proteins, including actinin and myosin were present in purified HMPV further supporting the involvement of the actin cytoskeleton in the formation of HMPV particles. Tubulin, vimentin and other cytoskeleton associated proteins were also detected suggesting their involvement in HMPV replication (S1 Table). Exosomal proteins were not found in high abundance in these fractions, suggesting that the purified fraction did not contain high levels of exosomes.

### HMPV-induced formation of intercellular extensions requires active actin dynamics

**Fig 4:**
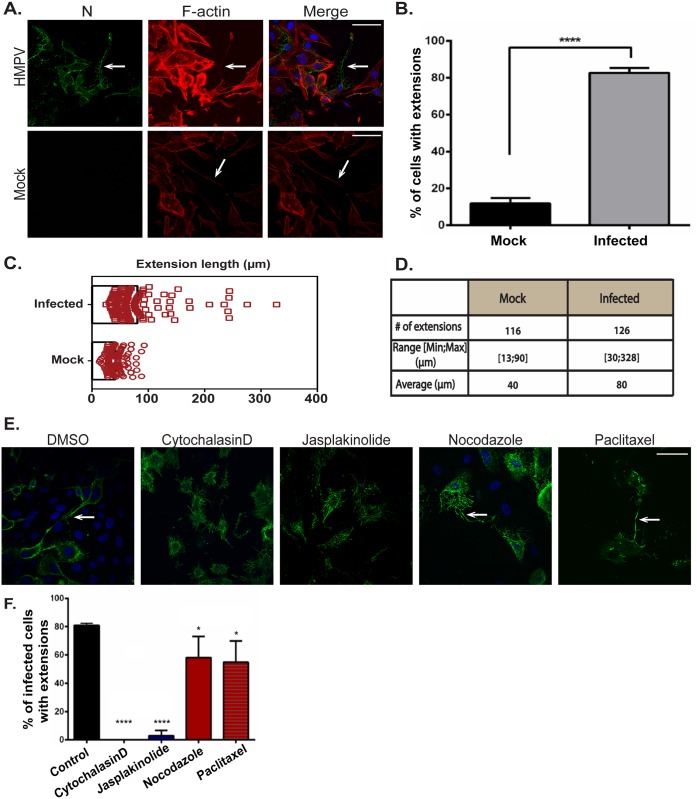
Formation of intercellular extensions requires active actin dynamics. (A) BEAS-2B cells were mock infected or inoculated with HMPV, and 24 h.p.i. cells were fixed and stained for HMPV N (green) or F-actin (red). Arrows indicate intercellular extensions. (B–D) BEAS-2B cells were either mock infected or infected with HMPV and 24 h.p.i. cells were fixed and processed for immunofluorescence staining. Images were taken, and 80–100 cells from three independent experiments were used to score the presence of extensions (B) or analyzed using the ImageJ analysis tool to determine extension length (C and D). (E) Cells were inoculated with HMPV for 2 hours and incubated with DMSO or the indicated drug, and 24 h.p.i. cells were stained with an antibody recognizing HMPV N (green). Images were acquired on a confocal microscope, and percentage of infected cells with extensions was manually determined for a total of 100 cells from three independent experiments. Scale bars = 50 μm. (F) Images were acquired on a confocal microscope and percentage of infected cells with extensions was manually determined for a total of 100 cells from three independent experiments. Data represent means ± SD. Statistical analysis was performed using one-way analysis of variance (ANOVA), (*** = *p*< 0.0001; ** = *p*< 0.001; * = *p*<0.05).

Intercellular extensions have been identified as a means of intercellular communication in different cell types, and research over the past decade has provided important information on their nature and function [68,69]. Primary human bronchial epithelial cells can form actin and microtubule-containing bridges between individual cells which vary greatly in length (range 50 μm–1 mm) and diameter (1 μm–20 μm) [70]. To determine whether the intercellular extensions observed in HMPV-infected BEAS-2B cells were induced or altered by HMPV infection, mock infected cells were stained for F-actin. Intercellular extensions were seen in uninfected cells (Fig 4A); however, the percentage of cells with intercellular extensions significantly increased upon HMPV infection, indicating that while these cellular structures can exist in uninfected cells, HMPV infection strongly induces their formation (Fig 4B). In addition, quantification of the length of intercellular extensions in uninfected- and HMPV-infected cells showed that the majority of extensions in infected cells were longer, with the average length in HMPV-infected cells (80 μm) being double that in uninfected cells (40 μm) (Fig 4C and 4D). To investigate the role of actin and microtubules in the formation of these intercellular extensions, inhibitors of cytoskeleton dynamics were added 2 hours after HMPV infection and left on cells until fixation at 24 h.p.i. The formation of intercellular extensions was slightly decreased but not blocked by the addition of nocodazole or paclitaxel, indicating that microtubule dynamics are not required for the formation of these structures (Fig 4E, arrows). In contrast, inhibition of actin polymerization by cytochalasin D and stabilization of actin by jasplakinolide significantly reduced formation of intercellular extensions in infected cells (Fig 4E and 4F). Thus, though both tubulin and F-actin are present in intercellular extensions, only actin polymerization is essential for intercellular extension formation. Collectively, these results indicate that HMPV infection leads to actin reorganization to induce formation and elongation of intercellular extensions.

### Rho GTPases involved in actin remodeling are important for the formation of actin-based structures induced by HMPV infection

**Fig 5:**
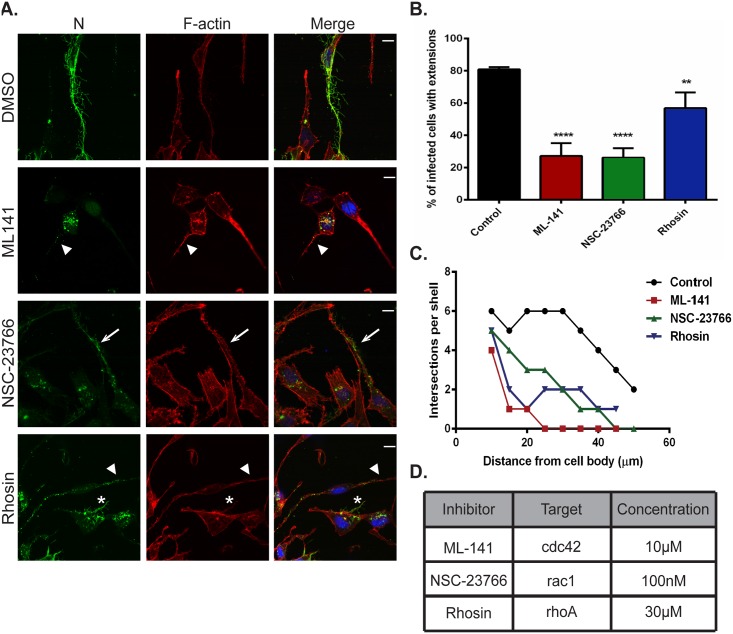
Rho GTPases Cdc42, Rac1 and RhoA contribute to formation of branched filamentous networks and intercellular extensions. (A) BEAS-2B cells were inoculated with HMPV, and the indicated drug was added 2 hours later. Twenty-four h.p.i. cells were fixed and stained for HMPV N or F-actin. Arrows indicate an intercellular extension. Arrowheads show punctate localization of N, and asterisks indicate short branched filaments. (B) Images acquired from three independent experiments were processed, and a total of 100 infected cells were manually assessed for the presence of extensions. Data represents mean ± SD. Statistical analysis was performed using one-way ANOVA, (*** = *p*< 0.0001; ** = *p*< 0.001). (C) A total of 10 cells from three independent experiments were traced using the NeuronJ plugin of the ImageJ analysis tool and the degree of branching was evaluated using Sholl analysis. (D) Table showing the targets of the inhibitors used and their specific concentration.

The actin cytoskeleton is highly dynamic and under the control of complex signaling pathways involving three main Rho family GTPases, Cdc42, Rac1 and RhoA. Activation of these proteins results in formation of protrusive filopodia, lamellipodia and stress fibers, respectively [71,72]. To address the potential role of these signaling pathways in the co-opting of the actin cytoskeleton during HMPV infection, we utilized cell permeable, targeted inhibitors for these GTPases and assessed the effects on the budding of branched filamentous networks and on formation of intercellular extensions. DMSO or inhibitors were added to cells 2 h.p.i., and 22 hours later cells were fixed and stained for F-actin to visualize changes in the actin cytoskeleton and the HMPV N protein. Addition of ML141, which primarily targets CDC42, resulted in a dramatic loss of filamentous structures at the cell periphery compared to control cells, and N was mostly cytosolic with faint staining in cellular extensions (Fig 5A, ML-141, arrowhead). In cells that were treated with NSC-23766, a targeted Rac1 inhibitor, N was localized in long intercellular extensions, but only short filaments were seen protruding from the main extension (Fig 5A, NSC-23766, arrow). Addition of Rhosin, an inhibitor of RhoA, led to a punctate localization pattern of N in intercellular extensions (Fig 5A, rhosin, arrowhead) and in some cells, short branched filaments were seen (Fig 5A, rhosin asterisk). These observations indicate that addition of any one of the Rho GTPase inhibitors does not result in dramatic loss of intercellular extension formation as was seen with disruption of actin dynamics (Fig 3); however each inhibitor resulted in a significant decrease in the percentage of infected cells with extensions compared to DMSO treated cells, with inhibitors targeting Cdc42 and Rac1 having a greater effect than the inhibitor which targets RhoA (Fig 5B). This suggests that induction of these extensions in HMPV-infected cells involves activation of the Rho family GTPase signaling pathways that control actin dynamics in the cell. Moreover, addition of the inhibitors targeting RhoA and Rac1 resulted in decreased but apparent filament formation and branching whereas addition of the Cdc42 inhibitor drastically decreased formation of branched filaments (Fig 5A), and these observations were confirmed by Sholl analysis (Fig 5C). Proteomic analysis of HMPV particles indicated the presence of Cdc42 and RhoA (S1 Table) further suggesting their importance for the HMPV replication cycle. While it is possible that the inhibitors also have effects beyond their target GTPase, our results suggest that coordination of activation of the three Rho GTPases, Rac1, Cdc42 and RhoA is involved in actin cytoskeleton rearrangement induced by HMPV to promote formation of intercellular extensions and budding of filamentous networks.

### P co-localizes with actin and induces formation of cellular extensions

**Fig 6:**
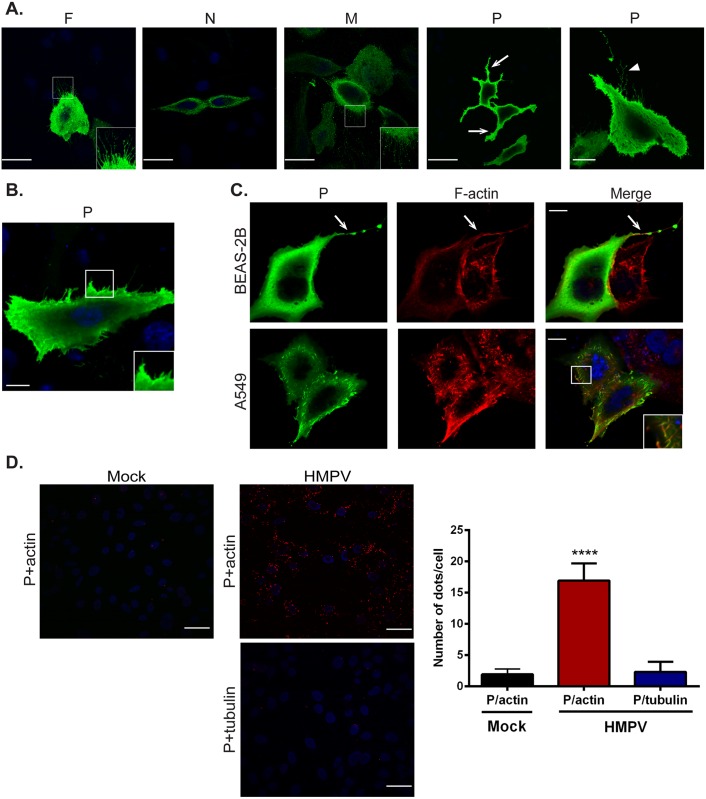
HMPV P induces formation of membrane extensions and co-localizes with actin. (A) BEAS-2B cells expressing HMPV F, N, M or P were processed for immunofluorescence and stained for the indicated viral protein (green) and DAPI (blue) to stain the nucleus. Insets show membrane extensions in cells transfected with F and M, arrows indicate long membrane extensions and the arrowhead shows a branched filament. (B) A549 cells were transfected with pCAGGS-HMPV P plasmid, and 24 hours post transfection, cells were fixed and processed for immunofluorescence staining. Inset shows membrane ruffling. (C) BEAS-2B or A549 cells were transfected with pCAGGS-HMPV P, and 24 hours later, cells were fixed and stained for P (green) and F-actin (red). Inset shows intracellular colocalization of P and F-actin, and arrows indicate colocalization of P and F-actin in a cellular extension. Scale bars = 10μm. (D) BEAS-2B cells were infected with HMPV, and 24 h.p.i, cells were fixed and proximity ligation assay was performed using the indicated antibodies. Each red signal denotes a reaction due to proximity of P and actin. Scale bars = 50μm. Quantification was done using BlobFinder software analysis tool. Graph shows mean ± SD. Statistical analysis was done using one-way ANOVA (*** = *p*< 0.0001).

The ability of enveloped viruses to bud infectious particles at cell membranes requires induction of membrane curvature and envelopment of viral components with a cell-derived membrane. For paramyxoviruses, membrane budding is driven principally by the M protein, at least partly due to its ability to bind and deform membranes, but efficiency can increase with addition of envelope proteins, N protein or accessory proteins [52,73]. To determine what viral proteins contribute to membrane remodeling during HMPV infection, we performed single transfections of N, P, M and F in BEAS-2B cells. Expression of the F or M proteins induced formation of short membrane extensions (Fig 6A insets), whereas N protein was primarily cytosolic, and no alterations of the membrane were observed with N expression. The HMPV M protein has been shown to bind lipid membranes and self-assemble into long helical filaments, and co-expression of both M and F induces filamentous VLP formation at the surface of Vero cells [18,21,74], consistent with our observation that F and M can induce membrane changes (Fig 6A). Interestingly, expression of the HMPV P protein in BEAS-2B cells induced changes to the plasma membrane and formation of membrane extensions, in contrast to what has been reported for other paramyxovirus P proteins (Fig 6A arrow). In addition, branched filaments were seen in some cells expressing P (Fig 6A arrowhead). Expression of P in A549 cells also resulted in deformation of the plasma membrane and induction of membrane ruffling (Fig 6B, inset). Immunostaining showed co-localization of P with F actin in both BEAS-2B (Fig 6C, arrow) and A549 cells (Fig 6C, inset). To further analyze the association between P and actin, we performed a proximity ligation assay, in which a positive signal requires two proteins in close proximity (<50nm). The percentage of cells with a positive proximity ligation signal for P and actin was significantly higher in HMPV-infected cells compared to mock infected cells (Fig 6D), indicating that HMPV P is in close proximity to actin, and thus likely either directly interacting with actin or with actin-associated proteins. As a control, proximity ligation for P and tubulin was performed, but no significant increase after HMPV infection was observed (Fig 6D). Paramyxovirus P proteins are major components of the viral replication complex and can interact with both N and the polymerase L [75]. Our results reveal a novel role for the HMPV P protein in induction of membrane extensions, suggesting the involvement of P in budding and egress of HMPV. This is also the first report of potential paramyxovirus phosphoprotein-actin or actin binding protein interaction, suggesting novel roles of P in HMPV exit from the cell.

### Intercellular extensions play a role in direct cell-to-cell spread of HMPV particles

**Fig 7:**
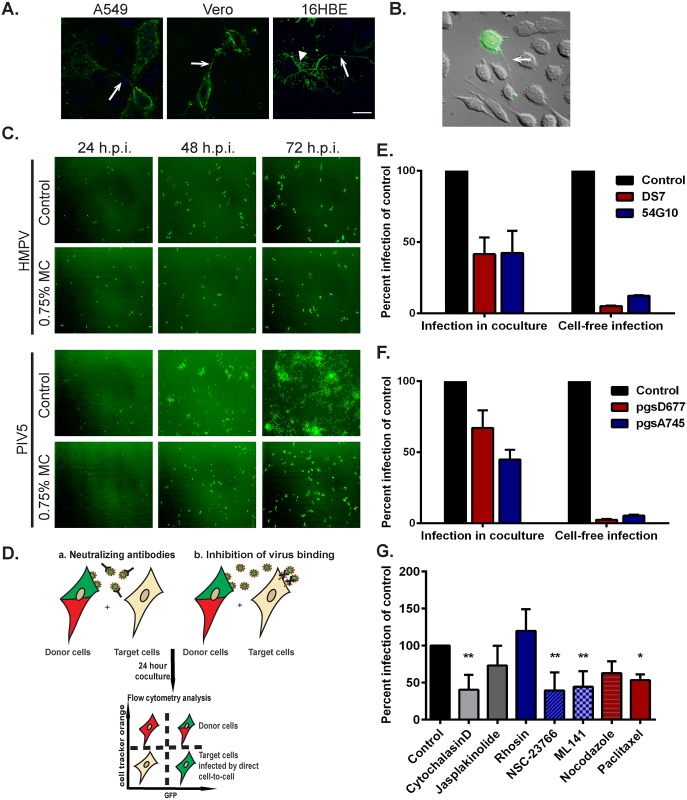
HMPV can spread directly from cell-to-cell. (A) A549, Vero or 16HBE cells were inoculated with HMPV, and 24 h.p.i., cells were fixed and stained for N protein. Arrows indicate intercellular extensions and arrowhead shows branched filaments. (B) BEAS-2B cells were inoculated with rgHMPV, and 24 h.p.i., cells were prepared for live imaging. Arrow indicates an intercellular extension. (C) BEAS-2B cells were infected with rgHMPV or rgPIV5 at an M.O.I. of 2. Six hours later, infection media was removed and replaced with regular media or media containing 0.75% methylcellulose (MC). Images were taken every 24 hours for 3 days. (D) Schematic of the coculture assay. BEAS-2B cells were inoculated with rgHMPV at an M.O.I. of 2. Forty-eight h.p.i., cells were stained with cell tracker CMRA orange dye for 30 minutes. Infected donor cells were then collected and added to naive target cells at a ratio of 1:1. To study direct cell transmission by blocking re-infection by released particles, the assay was done in the presence of neutralizing antibodies (E) or using target cells that lack a heparan sulfate binding factor for HMPV infection (F). Control represents infection without the presence of neutralizing antibodies (E) or with wt CHO cells (F). Twenty-four hours post coculture, cells were collected and analyzed by flow cytometry. (G) Cells were prepared for coculture assay as described in (D), and the indicated inhibitor was added at the time of mixing donor and infected cells and left in the media for 24 hours. Control represents addition of DMSO only. Data represent means ± SD for three independent experiments. One-way ANOVA was used for statistical analysis (*** = *p*< 0.0001; ** = *p*< 0.001; * = *p*<0.05).

To assess if intercellular extensions are generally associated with HMPV infection or are specific for BEAS-2B cells, other cell types including 16HBE, A549 and Vero cells were infected, and cells were immunostained for the HMPV N protein. Intercellular extensions containing HMPV viral proteins were present in each of these different cell types (Fig 7A), indicating that formation of these structures during HMPV infection is not restricted to BEAS-2B cells. Extensive networks of branching filaments were also seen in 16HBE cells (Fig 7A arrowhead), though not in Vero or A549 cells, suggesting that formation of these structures may be common to human bronchial epithelial cells. In addition, extensions were detected in live BEAS-2B cells infected with rgHMPV as seen in Fig 7B (arrow) extending from an infected cell to an uninfected cell, thus confirming that they are not artifacts of immunofluorescence processing and that they are associated with infection of HMPV in live cells. Extensions from infected cells were not always directed to the closest neighboring cells (Fig 7B) but can extend over long distances to reach another cell, consistent with our results that they can reach over 300 μm in length (Fig 4C). To initially examine the potential role of cell-cell spread in HMPV infection, we tested virus spread, seen as GFP expressing BEAS-2B cells (Fig 7C), in the presence or absence (control) of a viscous methyl cellulose overlay media to prevent diffusion of cell-free virus particles. The number of HMPV-infected cells increased by approximately two-fold from 24 to 48 hours in the presence or absence of methyl cellulose, with continuing increases at 72 h, and similar results were observed in four separate experiments, suggesting that HMPV can spread even when diffusion of released virus particles is compromised. In contrast, significant reduction in PIV5 spread was observed in the presence of methyl cellulose.

To verify that HMPV can spread directly from cell-to-cell, we developed a co-culture assay (Fig 7D). Cells were infected with rgHMPV (M.O.I. 2) for 48 hours and then stained with the cell tracker orange CMRA dye. The infected donor cells were then added to unstained target cells at a ratio of 1:1, and 24 hours post co-culture, cells were collected and analyzed by flow cytometry. GFP-only positive cells represented the newly infected target cells. To test direct cell-to-cell spread of HMPV, cells were co-cultured in the presence of two neutralizing antibodies, DS7 and 54G10 targeted against the fusion protein. These antibodies have been shown to inhibit infection by cell-free HMPV particles [76,77]. Consistent with this, pre-incubation of HMPV with DS7 or 54G10 prior to addition to cells significantly inhibited cell-free infection to around 10% that of control cells (Fig 7E, right panel). Approximately 60% of HMPV spread was inhibited by addition of neutralizing antibodies under co-culture conditions, indicating a reliance on the HMPV F protein. However, approximately 40% of spread remained in the presence of the antibodies (Fig 7E, left panel), suggesting that HMPV can spread in the presence of either DS7 or 54G10 and has a neutralizing antibody-independent mechanism of infection. This finding is consistent with a portion of HMPV infection of new target cells occurring via direct cell-to-cell spread through extensions.

Previous work in our laboratory demonstrated that binding and entry of HMPV require expression of heparan sulfate on the surface of target cells [17]. To determine whether spread of HMPV in the co-culture assay was dependent on heparan sulfate, we utilized CHO cell derivatives, psgD677 and pgsA745. pgsD677 cells are incapable of synthesizing heparan sulfate, while pgsA745 cells lack all glycosaminoglycans (GAGs) [47,78]. Consistent with previous studies, cell-free HMPV infection requires heparan sulfate (Fig 7F, right panel). However, HMPV-infected cells were able to efficiently spread infection to target cells that lack heparan sulfate under co-culture conditions (Fig 7F, left panel). Intercellular extensions can transfer cellular components between cells including cytoplasmic material, organelles, membrane proteins and signaling molecules [68,69,79]. These data combined with the results from the neutralizing antibodies strongly indicate that HMPV has two modes of infection: cell-free infection that is blocked by neutralizing antibodies and requires binding to heparan sulfate moieties, and direct cell-to-cell infection that is neutralizing antibody- and heparan sulfate independent.

We next determined the effect of the different cytoskeletal drugs, some of which interfered with the elongation of the extensions, on spread of HMPV in the presence of neutralizing antibodies. Infected donor cells were pretreated for one hour with the specific drug prior to incubation with naive target cells, and DS7 antibody was used for neutralization of released virus particles. While manipulation of microtubule dynamics by paclitaxel gave only small decreases in the number of cells with intercellular extensions (Fig 4E and 4F), paclitaxel treatment resulted in a significant decrease in intercellular spread of HMPV, suggesting a potential role for microtubules in spread separate from extension formation, though nocodazole treatment resulted in a reduction that was not statistically significant (Fig 7G). Disruption of actin polymerization with cytochalasin D, which dramatically decreased formation of intercellular extensions (Fig 4E and 4F), resulted in a significant decrease in HMPV spread, with the difference in level of inhibition potentially due to the addition time of the inhibitor post-infection. Inhibition of both Rac1 (NSC-7366) and Cdc42 (ML-141) significantly reduced cell-to-cell spread of HMPV (Fig 7G), consistent with the decrease in extensions observed with these inhibitors (Fig 5B). In contrast, inhibition of RhoA with the inhibitor rhosin did not influence intercellular spread of HMPV (Fig 7G), consistent with the minor effect of this inhibitor on extension formation (Fig 5B), and in contrast to reports that RhoA is important for RSV filament formation [61]. Thus, our results indicate that inhibition of actin polymerization, Cdc42 or Rac1, which results in significant decrease in the formation of intercellular extensions, also decreases intercellular spread of HMPV, supporting a role of the extensions in cell-to-cell spread of HMPV particles.

**Fig 8:**
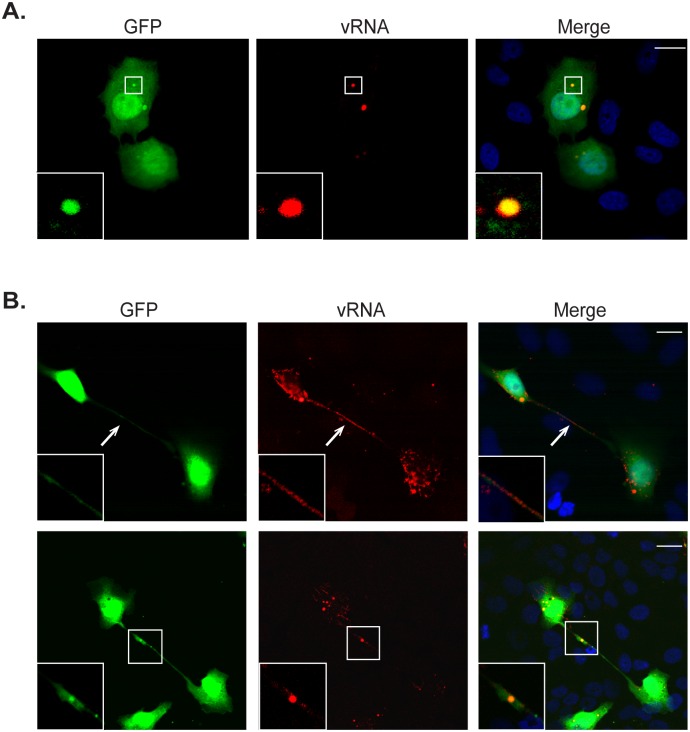
Viral RNA is present in intercellular extensions. (A) Twenty-four hours post infection with rgHMPV, BEAS-2B cells were fixed, permeabilized with 70% ethanol and incubated with the FISH probes targeting viral RNA overnight. Cells were then washed with 2xSCC buffer and mounted with vectashield. Inset indicates viral RNA in a potential inclusion body. (B) BEAS2B cells infected for 72 hours with rgHMPV were processed as in (A). Arrows indicate an intercellular extension and inset shows larger RNA-containing structure in intercellular extension.

To further examine the role of intercellular extensions in HMPV spread, FISH for detection of viral RNA was performed using 48 labeled probes targeted for vRNA (Fig 8A). BEAS-2B cells infected with rgHMPV were identified by GFP expression throughout the cytoplasm, though a localization of GFP in discrete punctate bodies was routinely seen. Viral RNA was detected in these discrete cytoplasmic structures (Fig 8A inset) that resemble the inclusion bodies where HMPV P localized (Fig 1A). Inclusion bodies have been detected for several paramyxoviruses and are thought to be sites of active RNA replication [56,80]. At 72 h.p.i., viral RNA was detected in intercellular extensions in cells infected with rgHMPV (Fig 8B arrow and inset). Interestingly, larger RNA containing structures were also seen in extensions, raising the possibility that nucleocapsids or bodies containing multiple genomes can be transported across intercellular extensions between cells (Fig 8B inset). The localization of the main HMPV structural proteins N, P, M and F (Fig 1A) and of viral RNA in intercellular extensions provides additional evidence for the involvement of extensions in HMPV spread.

## Discussion

As a recently discovered virus [7], several aspects of the HMPV replication cycle are poorly understood. In this study we reveal two distinct features of late stages of HMPV infection that contrast with what is known about paramyxovirus assembly and spread. Similar to other respiratory viruses, we show that HMPV can form filamentous structures at the surface of infected cells; however, the network of branched filamentous structures containing major HMPV proteins are more complex than what has been previously reported for respiratory viruses. The formation of these structures was dependent on actin polymerization and on actin associated signaling involving Rho GTPases Cdc42, Rac1 and RhoA. In addition, we provide evidence for a novel mode of HMPV transmission from cell-to-cell across actin-based intercellular extensions that occurs independent of heparan sulfate and neutralizing antibodies.

Formation of filamentous virus particles has been reported for several respiratory viruses including RSV and influenza virus [59,81–83]; however how these structures are assembled and their significance for viral infectivity is not well understood. For influenza viruses, the filamentous morphology of the virus depended on actin polymerization [59]. For RSV, actin and tubulin were not required for assembly of viral filaments, but actin was involved in anchoring the filaments to the cell surface and for virus replication [84]. HMPV has been shown to form filaments in infected LLC-MK2 cells [21,22]. Here we show that HMPV assembles into a complex network of branched filaments at the surface of BEAS-2B cells (Fig 1A), and these structures were also seen in 16HBE cells (Fig 7A) indicating that these are specific features of HMPV infection in bronchial epithelial cells. The formation of cell-associated branched networks in HMPV infected cells raises questions about their importance for HMPV infection. Electron microscopic images of purified HMPV show pleomorphic particles with both approximately spherical morphology and also filamentous forms [85]. Thus, whether the filamentous morphology is maintained after release of virus particles from BEAS-2B cells is not known. Recent electron tomography images of the closely related RSV show that the position of M protein under the plasma membrane drives the filamentous morphology of the particles, but virus release coincides with M no longer forming a layer under the plasma membrane and loss of the filamentous form [62]. In addition, our findings that branched filaments were seen in human bronchial epithelial cells and not in other cell types (Figs 1A and 7A) indicate an effect of cell origin on virus assembly.

The extensive remodeling of the plasma membrane and the cortical actin underneath involves manipulation of several factors that act to control cell shape at the plasma membrane, namely the actin cytoskeleton and Rho GTPase signaling controlling actin. Budding of the filamentous networks was dependent on actin polymerization and not on microtubules (Fig 3); however the initial assembly of filamentous structures that can deform membranes does not depend on actin dynamics. HMPV M has been shown to self-assemble into higher order structures, forming flexible helical filaments upon binding to lipids, and it is thought that the dimer subunits of M can associate through different side-by-side interactions which influence the curvature of the matrix arrays and thus virus morphogenesis [74]. In addition, HMPV P protein can form tetramers [86]; thus it is possible that self-oligomerization and assembly of viral proteins or association of viral proteins with cellular factors can drive the formation of the initial filaments irrespective of actin polymerization, but polymerization of actin is needed to further drive the assembly and budding of the extensive branched filaments. However, after treatment with cytocholasin D or latrunculin A, actin was still seen in close proximity with the filaments containing viral proteins (Fig 3A), suggesting actin may play an accessory role. Our results also indicate that the Rho GTPases involved in controlling actin dynamics and structure in the cell, Cdc42, Rac1 and RhoA, play an important role in the production of branched filaments (Fig 5A and 5C). Cdc42 is likely required for the assembly of filaments since addition of a Cdc42 inhibitor abolished formation of filaments in HMPV infected cells. Shorter filamentous structures (Fig 5A) and decreased filamentous branching (Fig 5C) were seen in cells treated with inhibitors of Rac1 and RhoA, suggesting a role for these GTPases in promoting further budding of the filaments but not necessarily in the initial assembly. One of the common downstream effectors of Rac1 and Cdc42 is the Arp2/3 initiation complex which activates actin polymerization as well as formation of branched actin filaments, so the role of Arp2/3 still needs to be investigated. While filaments were seen at the cell surface at 18 h.p.i., the formation of the extensive branched filamentous networks in infected cells occurred later during infection starting at 24 h.p.i. and developed further at 48 h.p.i. This suggests that the actin remodeling associated with these cellular changes was mediated by an accumulation of viral products.

Viruses have the ability to manipulate the actin cytoskeleton by encoding viral proteins that can directly bind actin or actin binding proteins or upstream mediators of actin signaling. Paramyxovirus proteins that were shown to bind actin include the M proteins of Sendai virus and NDV [87,88]. Here we provide the first example of a paramyxovirus P protein that induces changes to the plasma membrane and co-localizes with actin, most likely due to an association with actin or actin-associated proteins (Fig 6A). Recently, it has been shown that paramyxovirus P proteins can have roles beyond regulating viral RNA synthesis. The P protein of HPIV3 was found to bind SNAP29 protein and inhibit autophagosomal degradation to enhance release of virus particles [89]. Paramyxovirus P proteins are characterized by intrinsically disordered regions that allow interactions with multiple partners during the course of infection [90], and recent structural analysis of HMPV P was consistent with the presence of intrinsically disordered regions at the N- and C-termini [91]. It is possible that binding of P to different cellular partners during the course of infection may play a role in regulating its function during the infection cycle from viral RNA synthesis at early stages of infection to inducing plasma membrane deformation and possibly contributing to HMPV exit from the cell at the end of the replication cycle. Our proteomic analysis of purified HMPV particles (S1 Table) revealed the presence of actin nucleating proteins, filamin A and alpha-actinin as well as ezrin, radixin and moesin (ERM) proteins. HMPV P binding to any of these cellular proteins or activation of signaling pathways upstream of actin polymerization state could lead to the observed changes. It is also possible that as the global concentration of P in the cell increases at late times after infection, mimicking levels seen in transfected cells, the effect of P on membrane deformation becomes more prominent. It remains to be determined if other paramyxovirus P proteins have similar roles in late stages of the infection cycle or if HMPV P is unique among the viral family.

An increasing number of reports show that some enveloped viruses can transmit from cell-to-cell, and between hosts, in novel ways beyond release of individual virus particles into the extracellular matrix [29,35,43,44,92–96]. Viral induction or modification of cellular structures to allow cell-to-cell spread is an intriguing theme of several recent studies, and our work supports a model in which HMPV infection induces intercellular extensions that are key elements in direct cell-to-cell spread of this respiratory virus. For paramyxoviruses, cell-to-cell spread of viruses independent of particle release occurs for measles virus across neuronal synapses and fusion pores in epithelial cells and for RSV through syncytia formation in cell culture [39–43]. Recently, intercellular spread independent of neutralizing antibodies was reported for PIV5 [44]. These studies indicate that mechanisms of paramyxovirus spread can vary greatly and shift the more generally accepted mode of spread by single particle release to more complex models. Direct cell-to-cell transmission of virus particles overcomes the rate limiting step of diffusion of particles across the extracellular space and also provides a means by which particles can be transferred that evades the immune response. Our data from the co-culture assay indicate that HMPV particles can spread in a neutralizing antibody independent manner, and that spread is reduced when formation of intercellular extensions is inhibited by disruption of actin polymerization or by Cdc42 and Rac1 inhibition (Fig 7E and 7G). Formation of plasma membrane extensions by actin polymerization is driven mainly by activation of Rac1 and Cdc42 and their downstream effectors, and several viruses, including HIV-1 and pseudorabies virus, induce actin-based cellular extensions by activating these signaling pathways [97,98]; however this is the first report of a paramyxovirus that depends on Rho GTPase signaling to induce actin-based cellular extensions for intercellular spread. In addition, microtubules played an important role in HMPV spread (Fig 7G). For several RNA viruses, microtubules are involved in transport of the RNP complex to assembly sites at the plasma membrane [99], but this remains to be investigated for HMPV.

**Fig 9:**
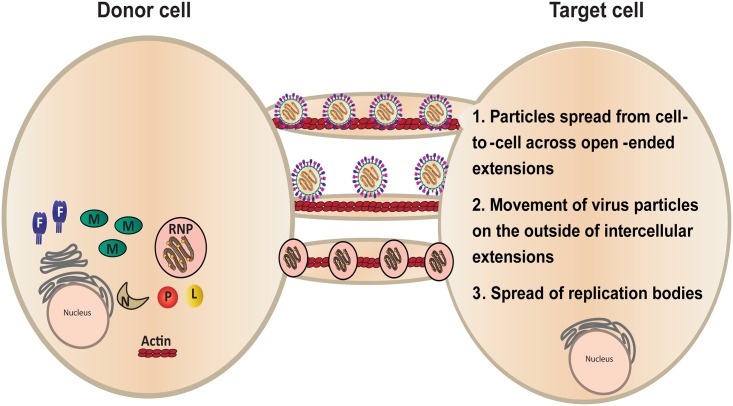
Models for cell-to-cell spread of HMPV in BEAS-2B cells. Virus particles exit an infected cell and move inside an open-ended intercellular extension to infect a target cell (model 1). In model 2, virus particles move from an infected cell to a target cell at the surface of an intercellular extension. Replication bodies can be transmitted from an infected cell to a target cell along an intercellular extension (model 3).

Several models currently exist for direct cell-to-cell spread of virus particles across cellular extensions [100] that could be applicable to our findings with HMPV. Open-ended or close-ended intercellular extensions could act as tunnels through which completely or partially assembled HMPV particles travel along actin filaments on the inside of the extension, allowing entry into the target cell (Fig 9, model 1), though the source of the viral membranes in this model remains unclear. Alternatively, budding of particles at the plasma membrane with subsequent movement of the particles across intercellular extensions from an infected cell to a donor cell could occur (Fig 9, model 2). This process, known as virus surfing, has been documented for several viruses including HIV-1 and MLV [34,101]. However, binding of virus to a cell surface receptor is required for entry and infection in this model, and our results demonstrate that infection of HMPV by direct cell-to-cell spread occurs independently of heparan sulfate which was shown to be an important binding factor for cell-free HMPV infection. Finally, it is possible that HMPV can spread infection directly from cell-to-cell by transfer of the RNP complex from an infected cell to a donor cell (Fig 9, model 3). Detection of vRNA by FISH analysis showed the presence of viral RNA and structures similar to replication bodies in an extension approaching another cell (Fig 8B). Spread of genetic material without formation or release of infectious virus particles has been suggested to occur for measles virus, both in neurons and in epithelial tissues [40,43]. The last two models of spread would protect the virus from neutralizing antibodies and bypass the need for receptor binding. Intercellular extensions have been shown to selectively transport cellular cargo from one cell to another [79], but further studies are needed to determine whether intercellular extensions utilized for HMPV spread are open or close ended.

## Supporting Information

S1 Table Proteomic Analysis of Purified HMPV Particles. (DOCX)

S2 Table Proteomic Analysis of Exosomes Purified from Mock Infected Cells. (DOCX)
