# Supplementary material for: Human metapneumovirus Induces Reorganization of the Actin Cytoskeleton for Direct Cell-to-Cell Spread
Source: PLoS Pathog. 2016 Sep 28;12(9):e1005922. doi: 10.1371/journal.ppat.1005922 (PMC5040343; doi:10.1371/journal.ppat.1005922)
Supplement: S1 Table — (DOCX) [file ppat.1005922.s001.docx]

| Protein  **Table S1. Proteomic identification of cellular proteins in ultrapurified HMPV particles.** | Accession # | # of peptides | # of MS/MS Spectra | Coverage (%) |
| --- | --- | --- | --- | --- |
| Actin, cytoplasmic 1 | P60709 | 24 | 40 | 66.93 |
| Actin, cytoplasmic 2 | P63261 | 24 | 40 | 66.93 |
| Keratin, type I cytoskeletal | P35527 | 27 | 34 | 65.33 |
| Keratin, type II cytoskeletal 1 | P04264 | 30 | 36 | 47.98 |
| 78 kDa glucose-regulated protein | P11021 | 34 | 41 | 53.67 |
| Tubulin beta chain | P07437 | 27 | 37 | 81.31 |
| Tubulin beta-4B chain | P68371 | 25 | 35 | 77.75 |
| Tubulin alpha-1B chain | P68363 | 25 | 31 | 75.83 |
| Tubulin alpha-1A chain | Q71U36 | 24 | 29 | 75.61 |
| Tubulin alpha-1C chain | Q9BQE3 | 24 | 29 | 75.95 |
| Tubulin beta-2B chain | Q9BVA1 | 21 | 30 | 65.84 |
| Tubulin beta-2A chain | Q13885 | 20 | 29 | 57.08 |
| Tubulin beta-4A chain | P04350 | 21 | 29 | 71.62 |
| Keratin, type I cytoskeletal 10 | P13645 | 23 | 27 | 44.52 |
| Annexin A2 | P07355 | 23 | 28 | 64.6 |
| Sodium/potassium-transporting ATPase subunit alpha-1 | P05023 | 23 | 25 | 32.94 |
| HLA class I histocompatibility antigen, A-1 alpha chain | P30443 | 17 | 28 | 49.32 |
| HLA class I histocompatibility antigen, A-36 alpha chain | P30455 | 17 | 28 | 49.32 |
| Voltage-dependent anion-selective channel protein 1 | P21796 | 15 | 17 | 71.38 |
| 4F2 cell-surface antigen heavy chain | P08195 | 18 | 23 | 34.6 |
| Endoplasmin | P14625 | 27 | 29 | 43.96 |
| HLA class I histocompatibility antigen, A-24 alpha chain | P05534 | 17 | 26 | 52.88 |
| HLA class I histocompatibility antigen, A-3 alpha chain | P04439 | 15 | 24 | 48.77 |
| HLA class I histocompatibility antigen, A-11 alpha chain | P13746 | 15 | 24 | 48.77 |
| Integrin beta-1 | P05556 | 23 | 28 | 35.84 |
| Serotransferrin | P02787 | 22 | 23 | 42.98 |
| Cytochrome b-c1 complex subunit 1, mitochondrial | P31930 | 17 | 19 | 47.5 |
| Tubulin alpha-3C/D chain | Q13748 | 18 | 22 | 59.56 |
| 60 kDa heat shock protein, mitochondrial | P10809 | 18 | 19 | 38.57 |
| Elongation factor Tu, mitochondrial | P49411 | 16 | 19 | 48.67 |
| POTE ankyrin domain family member E | Q6S8J3 | 9 | 17 | 8.09 |
| HLA class I histocompatibility antigen, A-23 alpha chain | P30447 | 15 | 24 | 49.04 |
| Calnexin | P27824 | 22 | 26 | 46.45 |
| Tubulin alpha-4A chain | P68366 | 16 | 21 | 46.43 |
| Tubulin beta-3 chain | Q13509 | 15 | 19 | 34.44 |
| Annexin A1 | P04083 | 15 | 18 | 49.13 |
| Hypoxia up-regulated protein 1 | Q9Y4L1 | 16 | 18 | 27.83 |
| HLA class I histocompatibility antigen, B-49 alpha chain | P30487 | 16 | 24 | 55.25 |
| HLA class I histocompatibility antigen, B-50 alpha chain | P30488 | 16 | 24 | 55.25 |
| HLA class I histocompatibility antigen, B-45 alpha chain | P30483 | 16 | 24 | 55.25 |
| Tubulin alpha-3E chain | Q6PEY2 | 13 | 15 | 47.56 |
| Neutral alpha-glucosidase AB | Q14697 | 17 | 23 | 32.1 |
| Protein disulfide-isomerase A3 | P30101 | 19 | 21 | 53.07 |
| Glyceraldehyde-3-phosphate dehydrogenase | P04406 | 15 | 18 | 64.78 |
| Putative annexin A2-like protein | A6NMY6 | 15 | 17 | 41.3 |
| Keratin, type II cytoskeletal 2 epidermal | P35908 | 14 | 14 | 37.09 |
| HLA class I histocompatibility antigen, B-15 alpha chain | P30464 | 13 | 21 | 43.37 |
| Pyruvate kinase PKM | P14618 | 12 | 14 | 38.04 |
| HLA class I histocompatibility antigen, B-58 alpha chain | P10319 | 12 | 18 | 44.2 |
| Putative HLA class I histocompatibility antigen, alpha chain | P01893 | 9 | 14 | 30.39 |
| Calreticulin | P27797 | 14 | 17 | 64.99 |
| HLA class I histocompatibility antigen, B-57 alpha chain | P18465 | 12 | 19 | 42.54 |
| HLA class I histocompatibility antigen, A-30 alpha chain | P16188 | 10 | 17 | 34.79 |
| HLA class I histocompatibility antigen, A-80 alpha chain | Q09160 | 9 | 15 | 24.93 |
| HLA class I histocompatibility antigen, B-53 alpha chain | P30491 | 12 | 18 | 43.37 |
| HLA class I histocompatibility antigen, B-35 alpha chain | P30685 | 12 | 18 | 43.37 |
| Fatty acid synthase | P49327 | 17 | 17 | 11.99 |
| Actin, alpha skeletal muscle | P68133 | 14 | 20 | 30.5 |
| Actin, alpha cardiac muscle 1 | P68032 | 14 | 20 | 30.5 |
| HLA class I histocompatibility antigen, B-46 alpha chain | P30484 | 12 | 19 | 41.71 |
| HLA class I histocompatibility antigen, B-56 alpha chain | P30495 | 12 | 19 | 41.71 |
| HLA class I histocompatibility antigen, B-52 alpha chain | P30490 | 11 | 17 | 48.07 |
| HLA class I histocompatibility antigen, B-47 alpha chain | P30485 | 13 | 21 | 42.54 |
| Tubulin alpha-8 chain | Q9NY65 | 11 | 14 | 33.41 |
| HLA class I histocompatibility antigen, B-44 alpha chain | P30481 | 12 | 19 | 40.06 |
| HLA class I histocompatibility antigen, B-40 alpha chain | Q04826 | 12 | 20 | 39.5 |
| HLA class I histocompatibility antigen, B-13 alpha chain | P30461 | 12 | 20 | 38.67 |
| Integrin alpha-3 | P26006 | 13 | 16 | 16.75 |
| Annexin A5 | P08758 | 13 | 14 | 52.81 |
| Elongation factor 1-alpha 1 | P68104 | 12 | 15 | 43.51 |
| HLA class I histocompatibility antigen, B-37 alpha chain | P18463 | 11 | 19 | 37.02 |
| HLA class I histocompatibility antigen, B-51 alpha chain | P18464 | 10 | 15 | 46.41 |
| HLA class I histocompatibility antigen, B-78 alpha chain | P30498 | 10 | 15 | 46.41 |
| HLA class I histocompatibility antigen, B-82 alpha chain | Q29718 | 12 | 19 | 41.44 |
| HLA class I histocompatibility antigen, B-59 alpha chain | Q29940 | 11 | 18 | 38.4 |
| HLA class I histocompatibility antigen, B-55 alpha chain | P30493 | 11 | 18 | 38.4 |
| ATP synthase subunit alpha, mitochondrial | P25705 | 10 | 11 | 28.39 |
| Annexin A6 | P08133 | 12 | 14 | 25.26 |
| HLA class I histocompatibility antigen, A-2 alpha chain | P01892 | 9 | 15 | 30.96 |
| HLA class I histocompatibility antigen, A-29 alpha chain | P30512 | 8 | 15 | 26.3 |
| Putative elongation factor 1-alpha-like 3 | Q5VTE0 | 11 | 14 | 38.53 |
| Cytochrome b-c1 complex subunit 2, mitochondrial | P22695 | 12 | 15 | 41.06 |
| Tubulin beta-6 chain | Q9BUF5 | 14 | 16 | 46.19 |
| HLA class I histocompatibility antigen, B-38 alpha chain | Q95365 | 10 | 17 | 35.36 |
| HLA class I histocompatibility antigen, B-39 alpha chain | P30475 | 10 | 17 | 35.36 |
| HLA class I histocompatibility antigen, B-67 alpha chain | Q29836 | 10 | 17 | 35.36 |
| HLA class I histocompatibility antigen, B-54 alpha chain | P30492 | 10 | 17 | 34.53 |
| Dolichyl-diphosphooligosaccharide--protein glycosyltransferase subunit 1 | P04843 | 13 | 14 | 32.78 |
| HLA class I histocompatibility antigen, A-69 alpha chain | P10316 | 9 | 15 | 30.14 |
| HLA class I histocompatibility antigen, B-27 alpha chain | P03989 | 10 | 17 | 35.36 |
| ATP synthase subunit beta, mitochondrial | P06576 | 11 | 13 | 34.78 |
| HLA class I histocompatibility antigen, Cw-12 alpha chain | P30508 | 8 | 12 | 29.23 |
| Protein disulfide-isomerase | P07237 | 16 | 16 | 40.55 |
| HLA class I histocompatibility antigen, A-32 alpha chain | P10314 | 7 | 12 | 25.48 |
| HLA class I histocompatibility antigen, A-74 alpha chain | P30459 | 7 | 12 | 25.48 |
| Actin, gamma-enteric smooth muscle | P63267 | 12 | 16 | 27.66 |
| Actin, aortic smooth muscle | P62736 | 12 | 16 | 27.59 |
| Cofilin-1 | P23528 | 9 | 10 | 62.65 |
| Stress-70 protein, mitochondrial | P38646 | 10 | 10 | 18.7 |
| Sodium/potassium-transporting ATPase subunit alpha-2 | P50993 | 8 | 9 | 12.16 |
| Sodium/potassium-transporting ATPase subunit alpha-3 | P13637 | 8 | 9 | 12.24 |
| HLA class I histocompatibility antigen, B-41 alpha chain | P30479 | 10 | 16 | 36.19 |
| Delta(24)-sterol reductase | Q15392 | 10 | 12 | 32.75 |
| HLA class I histocompatibility antigen, A-25 alpha chain | P18462 | 7 | 12 | 24.66 |
| HLA class I histocompatibility antigen, A-26 alpha chain | P30450 | 7 | 12 | 24.66 |
| HLA class I histocompatibility antigen, A-34 alpha chain | P30453 | 7 | 12 | 24.66 |
| HLA class I histocompatibility antigen, A-66 alpha chain | P30457 | 7 | 12 | 24.66 |
| HLA class I histocompatibility antigen, A-68 alpha chain | P01891 | 7 | 12 | 24.66 |
| HLA class I histocompatibility antigen, Cw-6 alpha chain | Q29963 | 7 | 11 | 26.23 |
| HLA class I histocompatibility antigen, Cw-7 alpha chain | P10321 | 6 | 11 | 22.68 |
| HLA class I histocompatibility antigen, Cw-15 alpha chain | Q07000 | 7 | 11 | 25.41 |
| Clathrin heavy chain 1 | Q00610 | 11 | 12 | 11.16 |
| HLA class I histocompatibility antigen, Cw-16 alpha chain | Q29960 | 6 | 10 | 21.86 |
| HLA class I histocompatibility antigen, A-31 alpha chain | P16189 | 6 | 11 | 20.82 |
| HLA class I histocompatibility antigen, A-43 alpha chain | P30456 | 6 | 11 | 20.82 |
| Cytoskeleton-associated protein 4 | Q07065 | 10 | 11 | 23.59 |
| HLA class I histocompatibility antigen, B-48 alpha chain | P30486 | 8 | 14 | 30.11 |
| Dolichyl-diphosphooligosaccharide--protein glycosyltransferase 48 kDa subunit | P39656 | 7 | 9 | 28.73 |
| Dolichyl-diphosphooligosaccharide--protein glycosyltransferase subunit 2 | P04844 | 8 | 9 | 26.15 |
| Adipocyte plasma membrane-associated protein | Q9HDC9 | 9 | 10 | 31.25 |
| Alpha-enolase | P06733 | 9 | 9 | 33.18 |
| HLA class I histocompatibility antigen, B-7 alpha chain | P01889 | 8 | 13 | 32.04 |
| HLA class I histocompatibility antigen, B-8 alpha chain | P30460 | 8 | 13 | 32.04 |
| HLA class I histocompatibility antigen, B-14 alpha chain | P30462 | 8 | 13 | 32.04 |
| HLA class I histocompatibility antigen, B-42 alpha chain | P30480 | 8 | 13 | 32.04 |
| Tubulin beta-8 chain | Q3ZCM7 | 8 | 11 | 19.37 |
| Tubulin beta-8 chain-like protein LOC260334 | A6NNZ2 | 8 | 11 | 19.37 |
| Alpha-actinin-4 | O43707 | 10 | 10 | 15.15 |
| HLA class I histocompatibility antigen, B-81 alpha chain | Q31610 | 7 | 12 | 28.45 |
| POTE ankyrin domain family member F | A5A3E0 | 6 | 9 | 5.49 |
| Succinate dehydrogenase [ubiquinone] flavoprotein subunit, mitochondrial | P31040 | 8 | 8 | 20.93 |
| ADP/ATP translocase 2 OS=Homo sapiens | P05141 | 11 | 11 | 33.89 |
| Beta-2-microglobulin OS=Homo sapiens | P61769 | 6 | 9 | 57.14 |
| Heat shock cognate 71 kDa protein | P11142 | 9 | 9 | 18.27 |
| HLA class I histocompatibility antigen, B-18 alpha chain | P30466 | 7 | 12 | 28.18 |
| Elongation factor 1-alpha 2 | Q05639 | 6 | 8 | 21.6 |
| Ezrin | P15311 | 9 | 11 | 18.77 |
| HLA class I histocompatibility antigen, Cw-17 alpha chain | Q95604 | 5 | 7 | 22.04 |
| Moesin | P26038 | 12 | 13 | 25.13 |
| Myosin-9 | P35579 | 8 | 8 | 6.99 |
| 3-hydroxyacyl-CoA dehydrogenase type-2 | Q99714 | 6 | 6 | 45.98 |
| Guanine nucleotide-binding protein G(I)/G(S)/G(T) subunit beta-1 | P62873 | 6 | 6 | 34.41 |
| Putative beta-actin-like protein 3 | Q9BYX7 | 4 | 7 | 10.93 |
| Transmembrane emp24 domain-containing protein 10 | P49755 | 7 | 8 | 42.47 |
| HLA class I histocompatibility antigen, A-33 alpha chain | P16190 | 5 | 8 | 21.37 |
| Prohibitin | P35232 | 7 | 8 | 35.66 |
| HLA class I histocompatibility antigen, B-73 alpha chain | Q31612 | 5 | 8 | 20.94 |
| HLA class I histocompatibility antigen, Cw-5 alpha chain | Q9TNN7 | 5 | 7 | 22.13 |
| HLA class I histocompatibility antigen, Cw-8 alpha chain | P30505 | 5 | 7 | 22.13 |
| ADP/ATP translocase 3 | P12236 | 8 | 8 | 30.2 |
| Thy-1 membrane glycoprotein | P04216 | 4 | 7 | 24.84 |
| HLA class I histocompatibility antigen, Cw-4 alpha chain | P30504 | 6 | 8 | 18.03 |
| Sideroflexin-1 | Q9H9B4 | 8 | 8 | 34.47 |
| EGF-like repeat and discoidin I-like domain-containing protein 3 | O43854 | 6 | 7 | 16.67 |
| HLA class I histocompatibility antigen, Cw-18 alpha chain | Q29865 | 6 | 8 | 18.03 |
| Galectin-1 | P09382 | 6 | 7 | 62.96 |
| Elongation factor 2 | P13639 | 7 | 7 | 13.29 |
| ADP/ATP translocase 1 | P12235 | 7 | 7 | 27.18 |
| Cytochrome b-c1 complex subunit Rieske, mitochondrial | P47985 | 7 | 8 | 36.5 |
| Unconventional myosin-Ic | O00159 | 6 | 6 | 8.84 |
| HLA class I histocompatibility antigen, Cw-14 alpha chain | P30510 | 5 | 7 | 15.03 |
| Voltage-dependent anion-selective channel protein 2 | P45880 | 6 | 6 | 28.23 |
| Calmodulin | P62158 | 6 | 6 | 72.48 |
| ATP synthase subunit d, mitochondrial | O75947 | 8 | 8 | 67.7 |
| Beta-actin-like protein 2 | Q562R1 | 5 | 8 | 11.97 |
| POTE ankyrin domain family member I | P0CG38 | 5 | 7 | 4 |
| Myosin light polypeptide 6 | P60660 | 4 | 6 | 34.44 |
| Protein S100-A11 | P31949 | 4 | 4 | 68.57 |
| Keratin, type I cytoskeletal 14 | P02533 | 7 | 7 | 13.56 |
| Putative cytochrome b-c1 complex subunit Rieske-like protein 1 | P0C7P4 | 6 | 7 | 27.56 |
| Golgi apparatus protein 1 | Q92896 | 4 | 4 | 4.66 |
| HLA class I histocompatibility antigen, alpha chain F | P30511 | 3 | 6 | 8.38 |
| Signal peptidase complex subunit 2 | Q15005 | 5 | 6 | 23.45 |
| Trifunctional enzyme subunit alpha, mitochondrial | P40939 | 6 | 6 | 11.4 |
| POTE ankyrin domain family member J | P0CG39 | 4 | 6 | 3.08 |
| Cytochrome c1, heme protein, mitochondrial | P08574 | 4 | 6 | 27.38 |
| HLA class I histocompatibility antigen, Cw-2 alpha chain | P30501 | 4 | 5 | 17.49 |
| Cytochrome b5 type B | O43169 | 4 | 5 | 40.41 |
| Alpha-actinin-1 | P12814 | 5 | 5 | 6.39 |
| Acetolactate synthase-like protein | A1L0T0 | 4 | 5 | 11.08 |
| 14-3-3 protein beta/alpha | P31946 | 6 | 6 | 28.05 |
| Transmembrane emp24 domain-containing protein 9 | Q9BVK6 | 5 | 6 | 20 |
| Fructose-bisphosphate aldolase A | P04075 | 5 | 5 | 20.33 |
| Heat shock-related 70 kDa protein 2 | P54652 | 5 | 5 | 10.33 |
| Prohibitin-2 | Q99623 | 6 | 6 | 28.09 |
| Protein disulfide-isomerase A6 | Q15084 | 5 | 5 | 21.14 |
| Heat shock protein HSP 90-beta | P08238 | 5 | 5 | 7.04 |
| ATP synthase-coupling factor 6, mitochondrial | P18859 | 3 | 3 | 44.44 |
| Hornerin | Q86YZ3 | 3 | 4 | 5.09 |
| Peptidyl-prolyl cis-trans isomerase A | P62937 | 7 | 7 | 58.18 |
| Glycerol-3-phosphate dehydrogenase, mitochondrial | P43304 | 5 | 5 | 8.8 |
| Guanine nucleotide-binding protein G(i) subunit alpha-2 | P04899 | 6 | 6 | 21.41 |
| ATP synthase subunit g, mitochondrial | O75964 | 4 | 5 | 34.95 |
| Hexokinase-1 | P19367 | 4 | 4 | 5.56 |
| Annexin A3 | P12429 | 4 | 4 | 15.17 |
| Radixin | P35241 | 6 | 7 | 11.32 |
| Cofilin-2 | Q9Y281 | 3 | 4 | 18.67 |
| EH domain-containing protein 1 | Q9H4M9 | 4 | 4 | 17.79 |
| Very long-chain specific acyl-CoA dehydrogenase, mitochondrial | P49748 | 4 | 4 | 7.94 |
| Epidermal growth factor receptor | P00533 | 3 | 3 | 4.38 |
| Beta-enolase | P13929 | 2 | 2 | 8.53 |
| Procollagen-lysine,2-oxoglutarate 5-dioxygenase 2 | O00469 | 5 | 6 | 9.5 |
| CD59 glycoprotein | P13987 | 4 | 6 | 25.78 |
| Voltage-dependent anion-selective channel protein 3 | Q9Y277 | 4 | 4 | 19.08 |
| Serpin H1 | P50454 | 6 | 6 | 25.6 |
| Cytochrome b-c1 complex subunit 6, mitochondrial | P07919 | 4 | 5 | 58.24 |
| Myosin-11 | P35749 | 3 | 3 | 2.54 |
| 5'-nucleotidase | P21589 | 4 | 4 | 10.28 |
| Sodium/potassium-transporting ATPase subunit alpha-4 | Q13733 | 4 | 4 | 6.9 |
| Keratin, type II cytoskeletal 6B | P04259 | 4 | 4 | 7.27 |
| Guanine nucleotide-binding protein G(I)/G(S)/G(T) subunit beta-2 | P62879 | 4 | 4 | 25.29 |
| DnaJ homolog subfamily B member 11 | Q9UBS4 | 5 | 5 | 21.51 |
| Transforming protein RhoA | P61586 | 5 | 6 | 38.34 |
| Keratin, type I cytoskeletal 17 | Q04695 | 5 | 5 | 9.26 |
| Histone H4 | P62805 | 4 | 4 | 39.81 |
| Filamin-A | P21333 | 5 | 5 | 2.04 |
| Erlin-1 | O75477 | 3 | 3 | 10.98 |
| Guanine nucleotide-binding protein G(k) subunit alpha | P08754 | 4 | 4 | 14.69 |
| Sideroflexin-3 | Q9BWM7 | 3 | 3 | 12.62 |
| Histone H2B type 1-H | Q93079 | 3 | 3 | 28.57 |
| Histone H2B type 2-F | Q5QNW6 | 3 | 3 | 28.57 |
| Histone H2B type 1-C/E/F/G/I | P62807 | 3 | 3 | 28.57 |
| Histone H2B type 1-D | P58876 | 3 | 3 | 28.57 |
| Histone H2B type 1-K | O60814 | 3 | 3 | 28.57 |
| Histone H2B type 1-L | Q99880 | 3 | 3 | 28.57 |
| Histone H2B type 1-M | Q99879 | 3 | 3 | 28.57 |
| Histone H2B type 1-N | Q99877 | 3 | 3 | 28.57 |
| Histone H2B type F-S | P57053 | 3 | 3 | 28.57 |
| Erythrocyte band 7 integral membrane protein | P27105 | 4 | 4 | 28.13 |
| CD97 antigen | P48960 | 4 | 4 | 6.95 |
| Glucosidase 2 subunit beta | P14314 | 5 | 5 | 13.26 |
| Heat shock protein beta-1 | P04792 | 4 | 4 | 38.54 |
| Potassium-transporting ATPase alpha chain 1 | P20648 | 2 | 2 | 2.71 |
| ATP synthase subunit gamma, mitochondrial | P36542 | 4 | 4 | 22.15 |
| Transmembrane protein 43 | Q9BTV4 | 5 | 5 | 17 |
| Protein disulfide-isomerase A4 | P13667 | 3 | 3 | 5.58 |
| Rho-related GTP-binding protein RhoC | P08134 | 4 | 5 | 32.12 |
| Phosphate carrier protein, mitochondrial | Q00325 | 5 | 5 | 14.64 |
| Sodium/potassium-transporting ATPase subunit beta-1 | P05026 | 5 | 5 | 23.76 |
| Erlin-2 | O94905 | 3 | 3 | 9.14 |
| Cytochrome b-c1 complex subunit 7 | P14927 | 3 | 3 | 27.03 |
| Ras-related protein Rab-10 | P61026 | 4 | 4 | 22.5 |
| 14-3-3 protein zeta/delta | P63104 | 5 | 5 | 20.82 |
| Keratin, type II cytoskeletal 5 | P13647 | 4 | 4 | 6.95 |
| HLA class I histocompatibility antigen, Cw-3 alpha chain | P04222 | 3 | 3 | 11.2 |
| HLA class I histocompatibility antigen, alpha chain E | P13747 | 3 | 4 | 12.01 |
| Tubulin alpha chain-like 3 | A6NHL2 | 4 | 5 | 4.71 |
| 60S ribosomal protein L6 | Q02878 | 2 | 2 | 11.11 |
| Brain acid soluble protein 1 | P80723 | 4 | 4 | 38.77 |
| Neutral cholesterol ester hydrolase 1 | Q6PIU2 | 3 | 3 | 11.76 |
| Histone H2B type 1-O | P23527 | 2 | 2 | 20.63 |
| Histone H2B type 1-B | P33778 | 2 | 2 | 20.63 |
| Histone H2B type 1-J | P06899 | 2 | 2 | 20.63 |
| Histone H2B type 2-E | Q16778 | 2 | 2 | 20.63 |
| Histone H2B type 3-B | Q8N257 | 2 | 2 | 20.63 |
| Heat shock 70 kDa protein 1-like O | P34931 | 3 | 3 | 6.24 |
| Neuroblast differentiation-associated protein AHNAK | Q09666 | 3 | 3 | 0.46 |
| Dihydropyrimidinase-related protein 2 | Q16555 | 1 | 1 | 2.8 |
| Mannosyl-oligosaccharide glucosidase | Q13724 | 4 | 4 | 10.75 |
| Myoferlin | Q9NZM1 | 5 | 5 | 3.49 |
| Keratin, type I cytoskeletal 16 | P08779 | 4 | 4 | 5.29 |
| Epoxide hydrolase 1 | P07099 | 4 | 4 | 10.99 |
| Protein S100-A10 | P60903 | 3 | 4 | 45.36 |
| Keratin, type II cytoskeletal 6C | P48668 | 3 | 3 | 5.5 |
| Keratin, type II cytoskeletal 6A | P02538 | 3 | 3 | 5.5 |
| Serine/threonine-protein phosphatase PP1-beta catalytic subunit | P62140 | 2 | 2 | 10.7 |
| Serine/threonine-protein phosphatase PP1-gamma catalytic subunit | P36873 | 2 | 2 | 10.84 |
| Serine/threonine-protein phosphatase PP1-alpha catalytic subunit | P62136 | 2 | 2 | 10.61 |
| Chloride intracellular channel protein 1 | O00299 | 4 | 4 | 19.92 |
| Niemann-Pick C1 protein | O15118 | 3 | 3 | 3.83 |
| Gamma-enolase | P09104 | 2 | 2 | 8.06 |
| 14-3-3 protein gamma | P61981 | 4 | 4 | 16.6 |
| Guanine nucleotide-binding protein G(i) subunit alpha-1 | P63096 | 3 | 3 | 10.17 |
| Pyruvate dehydrogenase E1 component subunit beta, mitochondrial | P11177 | 2 | 2 | 7.52 |
| Leucine-rich PPR motif-containing protein, mitochondrial | P42704 | 2 | 2 | 2.37 |
| Thioredoxin-related transmembrane protein 1 | Q9H3N1 | 3 | 3 | 13.21 |
| Sodium/potassium-transporting ATPase subunit beta-3 | P54709 | 4 | 4 | 22.22 |
| CAAX prenyl protease 1 homolog | O75844 | 3 | 3 | 7.16 |
| Transmembrane emp24 domain-containing protein 4 | Q7Z7H5 | 2 | 3 | 12.33 |
| Tubulin beta-1 chain | Q9H4B7 | 3 | 3 | 4.66 |
| Cytochrome c oxidase subunit 5A, mitochondrial | P20674 | 3 | 3 | 26.67 |
| 7-dehydrocholesterol reductase | Q9UBM7 | 2 | 2 | 4.42 |
| Large neutral amino acids transporter small subunit 1 | Q01650 | 2 | 2 | 3.16 |
| Putative tubulin-like protein alpha-4B | Q9H853 | 1 | 2 | 5.81 |
| Alpha-actinin-2 | P35609 | 3 | 3 | 4.03 |
| Guanine nucleotide-binding protein G(s) subunit alpha isoforms XLas | Q5JWF2 | 3 | 3 | 3.47 |
| Guanine nucleotide-binding protein G(s) subunit alpha isoforms short | P63092 | 3 | 3 | 9.14 |
| Ras-related protein Rab-8A | P61006 | 3 | 3 | 16.43 |
| ATP synthase subunit e, mitochondrial | P56385 | 2 | 2 | 30.43 |
| Tricarboxylate transport protein, mitochondrial | P53007 | 3 | 3 | 11.25 |
| Lon protease homolog, mitochondrial | P36776 | 2 | 2 | 5.01 |
| Thioredoxin domain-containing protein 5 | Q8NBS9 | 2 | 2 | 6.02 |
| Translocon-associated protein subunit delta | P51571 | 3 | 3 | 19.65 |
| Nucleoside diphosphate kinase B | P22392 | 2 | 2 | 20.39 |
| Triosephosphate isomerase | P60174 | 3 | 3 | 19.58 |
| Keratin, type I cytoskeletal 19 | P08727 | 3 | 3 | 4 |
| Keratin, type I cytoskeletal 15 | P19012 | 3 | 3 | 3.51 |
| HLA class I histocompatibility antigen, Cw-1 alpha chain | P30499 | 2 | 2 | 8.2 |
| Keratin, type I cytoskeletal 28 | Q7Z3Y7 | 3 | 3 | 5.39 |
| Ras-related protein Rab-1B | Q9H0U4 | 3 | 3 | 18.41 |
| Dolichyl-diphosphooligosaccharide--protein glycosyltransferase subunit STT3A | P46977 | 1 | 2 | 3.69 |
| Flotillin-1 | O75955 | 1 | 1 | 3.51 |
| Alkyldihydroxyacetonephosphate synthase, peroxisomal | O00116 | 2 | 2 | 5.47 |
| Putative endoplasmin-like protein | Q58FF3 | 2 | 2 | 4.76 |
| Potassium-transporting ATPase alpha chain 2 | P54707 | 2 | 2 | 2.12 |
| ATP synthase F(0) complex subunit B1, mitochondrial | P24539 | 3 | 3 | 12.5 |
| Translocation protein SEC62 | Q99442 | 3 | 3 | 7.77 |
| Surfeit locus protein 4 | O15260 | 2 | 2 | 11.52 |
| ATP synthase subunit O, mitochondrial | P48047 | 2 | 2 | 13.62 |
| Histone H3.2 | Q71DI3 | 2 | 2 | 28.68 |
| Inosine-5'-monophosphate dehydrogenase 2 | P12268 | 2 | 2 | 11.87 |
| Calcium-binding mitochondrial carrier protein Aralar2 | Q9UJS0 | 1 | 1 | 3.26 |
| Creatine kinase B-type | P12277 | 1 | 1 | 4.46 |
| Profilin-1 | P07737 | 3 | 3 | 26.43 |
| Stomatin-like protein 2, mitochondrial | Q9UJZ1 | 3 | 3 | 11.8 |
| Heat shock 70 kDa protein 6 | P17066 | 3 | 3 | 4.98 |
| Keratin, type II cytoskeletal 8 | P05787 | 2 | 2 | 4.35 |
| 3-ketodihydrosphingosine reductase | Q06136 | 2 | 2 | 13.55 |
| 14-3-3 protein theta | P27348 | 4 | 4 | 20.82 |
| Putative nucleoside diphosphate kinase | O60361 | 1 | 1 | 13.87 |
| Glucose-6-phosphate isomerase | P06744 | 1 | 1 | 2.69 |
| 14-3-3 protein epsilon | P62258 | 3 | 3 | 14.51 |
| 14-3-3 protein sigma | P31947 | 3 | 3 | 16.94 |
| Transgelin-2 | P37802 | 1 | 1 | 9.05 |
| Keratin, type II cytoskeletal 4 | P19013 | 2 | 2 | 3.75 |
| ADP-ribosylation factor 3 | P61204 | 1 | 2 | 9.94 |
| ADP-ribosylation factor 1 | P84077 | 1 | 2 | 9.94 |
| Solute carrier family 2, facilitated glucose transporter member 1 | P11166 | 2 | 2 | 5.49 |
| Heat shock protein HSP 90-alpha | P07900 | 2 | 2 | 3.55 |
| Trophoblast glycoprotein | Q13641 | 1 | 1 | 3.33 |
| Guanine nucleotide-binding protein G(I)/G(S)/G(O) subunit gamma-12 | Q9UBI6 | 2 | 2 | 34.72 |
| Ras-related C3 botulinum toxin substrate 1 | P63000 | 4 | 4 | 34.9 |
| Isocitrate dehydrogenase [NADP], mitochondrial | P48735 | 1 | 1 | 2.88 |
| Heterogeneous nuclear ribonucleoprotein A0 | Q13151 | 1 | 1 | 5.25 |
| Actin-related protein 2/3 complex subunit 1B | O15143 | 1 | 1 | 4.84 |
| ADP/ATP translocase 4 | Q9H0C2 | 2 | 2 | 6.35 |
| GPI-anchor transamidase | Q92643 | 2 | 2 | 5.57 |
| Guanine nucleotide-binding protein G(o) subunit alpha | P09471 | 2 | 2 | 6.5 |
| Lysosome-associated membrane glycoprotein 1 | P11279 | 2 | 2 | 7.67 |
| Ras GTPase-activating-like protein IQGAP1 | P46940 | 1 | 1 | 1.09 |
| Pyrroline-5-carboxylate reductase 2 | Q96C36 | 1 | 1 | 4.06 |
| Pyrroline-5-carboxylate reductase 1, mitochondrial | P32322 | 1 | 1 | 4.08 |
| Keratin, type II cytoskeletal 75 | O95678 | 1 | 1 | 2.18 |
| Keratin, type II cytoskeletal 79 | Q5XKE5 | 1 | 1 | 2.24 |
| Keratin, type I cytoskeletal 12 | Q99456 | 2 | 2 | 1.82 |
| ATPase family AAA domain-containing protein 3A | Q9NVI7 | 1 | 1 | 2.21 |
| ATPase family AAA domain-containing protein 3C | Q5T2N8 | 1 | 1 | 3.41 |
| Keratin, type II cytoskeletal 1b | Q7Z794 | 1 | 1 | 2.08 |
| Acetyl-CoA acetyltransferase, mitochondrial | P24752 | 1 | 1 | 3.98 |
| Keratin, type I cytoskeletal 25 | Q7Z3Z0 | 2 | 2 | 4 |
| Keratin, type I cytoskeletal 27 | Q7Z3Y8 | 2 | 2 | 3.92 |
| CD9 antigen | P21926 | 2 | 2 | 15.35 |
| 10 kDa heat shock protein, mitochondrial | P61604 | 1 | 1 | 13.73 |
| Annexin A4 | P09525 | 2 | 2 | 8.46 |
| Multifunctional protein ADE2 | P22234 | 1 | 1 | 8 |
| Aminopeptidase N | P15144 | 1 | 1 | 1.65 |
| Peroxisomal multifunctional enzyme type 2 | P51659 | 1 | 1 | 2.04 |
| Dolichol-phosphate mannosyltransferase subunit 1 | O60762 | 2 | 2 | 6.92 |
| Myosin-14 | Q7Z406 | 1 | 1 | 0.8 |
| NADH dehydrogenase [ubiquinone] 1 alpha subcomplex subunit 5 | Q16718 | 1 | 1 | 22.41 |
| Microsomal glutathione S-transferase 1 | P10620 | 1 | 1 | 7.74 |
| Eukaryotic initiation factor 4A-I | P60842 | 2 | 2 | 4.93 |
| Neuroplastin | Q9Y639 | 2 | 2 | 8.29 |
| Alpha-actinin-3 | Q08043 | 1 | 1 | 1.44 |
| Filamin-B | O75369 | 1 | 1 | 0.42 |
| Filamin-C | Q14315 | 1 | 1 | 0.4 |
| Isocitrate dehydrogenase [NAD] subunit alpha, mitochondrial | P50213 | 1 | 1 | 4.64 |
| Endoplasmic reticulum resident protein 29 | P30040 | 1 | 1 | 9.2 |
| Heat shock 70 kDa protein 1A/1B | P08107 | 2 | 2 | 3.74 |
| Putative heat shock 70 kDa protein 7 | P48741 | 2 | 2 | 6.54 |
| Ras-related protein Rab-1A | P62820 | 2 | 2 | 10.73 |
| Ras-related protein Rab-35 | Q15286 | 2 | 2 | 10.95 |
| Ras-related protein Rab-8B | Q92930 | 2 | 2 | 10.63 |
| Ras-related protein Rab-15 | P59190 | 2 | 2 | 10.38 |
| Putative Ras-related protein Rab-1C | Q92928 | 2 | 2 | 10.95 |
| Sulfide:quinone oxidoreductase, mitochondrial | Q9Y6N5 | 2 | 2 | 8 |
| Endoplasmic reticulum resident protein 44 | Q9BS26 | 1 | 1 | 2.96 |
| Dihydrolipoyllysine-residue acetyltransferase component of pyruvate dehydrogenase complex, mitochondrial | P10515 | 1 | 1 | 2.32 |
| Charged multivesicular body protein 4b | Q9H444 | 1 | 1 | 6.25 |
| Transmembrane 9 superfamily member 4 | Q92544 | 1 | 1 | 2.18 |
| Barrier-to-autointegration factor | O75531 | 1 | 1 | 13.48 |
| Vesicular integral-membrane protein VIP36 | Q12907 | 2 | 2 | 15.17 |
| Unconventional myosin-Ib | O43795 | 2 | 2 | 3.52 |
| Electron transfer flavoprotein subunit alpha, mitochondrial | P13804 | 1 | 1 | 8.11 |
| Fructose-bisphosphate aldolase C | P09972 | 1 | 1 | 7.69 |
| Transmembrane protein 109 | Q9BVC6 | 1 | 1 | 4.94 |
| Pituitary tumor-transforming gene 1 protein-interacting protein O | P53801 | 1 | 1 | 10 |
| Latrophilin-2 | O95490 | 2 | 2 | 3.56 |
| Zinc transporter ZIP14 | Q15043 | 1 | 1 | 4.07 |
| Trifunctional enzyme subunit beta, mitochondrial | P55084 | 1 | 1 | 1.9 |
| Dolichol-phosphate mannosyltransferase subunit 3 | Q9P2X0 | 1 | 1 | 13.04 |
| Cytochrome c oxidase subunit 7C, mitochondrial | P15954 | 1 | 1 | 26.98 |
| Glial fibrillary acidic protein | P14136 | 1 | 1 | 2.55 |
| Keratin, type II cytoskeletal 7 | P08729 | 1 | 1 | 2.35 |
| Keratin, type II cytoskeletal 80 | Q6KB66 | 1 | 1 | 2.43 |
| Protein tweety homolog 3 | Q9C0H2 | 1 | 1 | 2.68 |
| Tapasin | O15533 | 3 | 3 | 10.49 |
| NADH-cytochrome b5 reductase 3 | P00387 | 1 | 1 | 6.31 |
| Histone H2A type 1-B/E | P04908 | 1 | 1 | 14.62 |
| Histone H2A type 1-C | Q93077 | 1 | 1 | 14.62 |
| Histone H2A type 1-D | P20671 | 1 | 1 | 14.62 |
| Histone H2A type 1-H | Q96KK5 | 1 | 1 | 14.84 |
| Histone H2A type 1-J | Q99878 | 1 | 1 | 14.84 |
| Histone H2A type 1 | P0C0S8 | 1 | 1 | 14.62 |
| Histone H2A type 2-A | Q6FI13 | 1 | 1 | 14.62 |
| Histone H2A type 2-C | Q16777 | 1 | 1 | 14.73 |
| Histone H2A type 3 | Q7L7L0 | 1 | 1 | 14.62 |
| Histone H2A.J | Q9BTM1 | 1 | 1 | 14.73 |
| ATP synthase subunit f, mitochondrial | P56134 | 1 | 1 | 11.7 |
| Destrin | P60981 | 1 | 1 | 7.88 |
| Torsin-1A-interacting protein 1 | Q5JTV8 | 1 | 1 | 2.06 |
| L-lactate dehydrogenase B chain | P07195 | 2 | 2 | 9.28 |
| Rho GDP-dissociation inhibitor 1 | P52565 | 1 | 1 | 7.35 |
| Prosaposin | P07602 | 1 | 1 | 2.86 |
| Histone H3.1 | P68431 | 2 | 2 | 28.68 |
| Peptidyl-prolyl cis-trans isomerase B | P23284 | 1 | 1 | 6.02 |
| Clathrin heavy chain 2 | P53675 | 1 | 1 | 1.04 |
| 14-3-3 protein eta | Q04917 | 2 | 2 | 7.32 |
| Inhibitor of nuclear factor kappa-B kinase-interacting protein | Q70UQ0 | 2 | 2 | 10.29 |
| Inverted formin-2 | Q27J81 | 1 | 1 | 1.12 |
| CD151 antigen | P48509 | 2 | 2 | 5.93 |
| Calmegin | O14967 | 1 | 2 | 1.97 |
| Keratin, type II cytoskeletal 2 oral | Q01546 | 2 | 2 | 2.82 |
| Ras-related C3 botulinum toxin substrate 3 | P60763 | 2 | 2 | 19.79 |
| Heterogeneous nuclear ribonucleoprotein K | P61978 | 1 | 1 | 2.59 |
| Succinate dehydrogenase [ubiquinone] iron-sulfur subunit, mitochondrial | P21912 | 1 | 1 | 3.93 |
| Vimentin | P08670 | 1 | 1 | 3 |
| Heat shock protein 75 kDa, mitochondrial | Q12931 | 1 | 1 | 1.99 |
| Myosin regulatory light chain 12B | O14950 | 1 | 1 | 5.81 |
| Myosin regulatory light chain 12A | P19105 | 1 | 1 | 5.85 |
| Enoyl-CoA hydratase, mitochondrial | P30084 | 1 | 1 | 5.86 |
| Guanine nucleotide-binding protein subunit alpha-12 | Q03113 | 1 | 1 | 2.89 |
| Guanine nucleotide-binding protein subunit alpha-13 | Q14344 | 1 | 1 | 2.92 |
| Guanine nucleotide-binding protein G(olf) subunit alpha | P38405 | 1 | 1 | 2.89 |
| Guanine nucleotide-binding protein G(t) subunit alpha-1 | P11488 | 1 | 1 | 3.14 |
| Guanine nucleotide-binding protein G(t) subunit alpha-2 | P19087 | 1 | 1 | 3.11 |
| Guanine nucleotide-binding protein G(t) subunit alpha-3 | A8MTJ3 | 1 | 1 | 3.11 |
| GTP-binding protein SAR1a | Q9NR31 | 1 | 1 | 10.1 |
| Proteasome activator complex subunit 1 | Q06323 | 1 | 1 | 4.82 |
| Eukaryotic initiation factor 4A-II | Q14240 | 1 | 1 | 2.46 |
| Lysosome membrane protein 2 | Q14108 | 1 | 1 | 6.28 |
| Copine-1 | Q99829 | 1 | 1 | 3.72 |
| Magnesium transporter protein 1 | Q9H0U3 | 1 | 1 | 2.99 |
| Pyruvate dehydrogenase E1 component subunit alpha, somatic form, mitochondrial | P08559 | 1 | 2 | 3.59 |
| Pyruvate dehydrogenase E1 component subunit alpha, testis-specific form, mitochondrial | P29803 | 1 | 2 | 3.61 |
| Transmembrane protein 205 | Q6UW68 | 1 | 1 | 14.81 |
| Peroxiredoxin-1 | Q06830 | 1 | 1 | 5.53 |
| Peroxiredoxin-2 | P32119 | 1 | 1 | 5.56 |
| Myosin light chain 6B | P14649 | 1 | 1 | 6.25 |
| Signal recognition particle receptor subunit alpha | P08240 | 1 | 1 | 1.57 |
| Cell division control protein 42 homolog | P60953 | 2 | 2 | 14.66 |
| Ras-related protein Rab-37 | Q96AX2 | 1 | 1 | 4.93 |
| Ras-related protein Rab-33B | Q9H082 | 1 | 1 | 4.8 |
| Ras-related protein Rab-12 | Q6IQ22 | 1 | 1 | 4.51 |
| Ras-related protein Rab-14 | P61106 | 1 | 1 | 5.12 |
| Ras-related protein Rab-30 | Q15771 | 1 | 1 | 5.42 |
| Ras-related protein Rab-3A | P20336 | 1 | 1 | 5 |
| Ras-related protein Rab-3B | P20337 | 1 | 1 | 5.02 |
| Ras-related protein Rab-3C | Q96E17 | 1 | 1 | 4.85 |
| Ras-related protein Rab-3D | O95716 | 1 | 1 | 5.02 |
| Ras-related protein Rab-43 | Q86YS6 | 1 | 1 | 5.19 |
| Ras-related protein Rab-4B | P61018 | 1 | 1 | 5.16 |
| Ras-related protein Rab-39B | Q96DA2 | 1 | 1 | 5.16 |
| Ras-related protein Rab-6A | P20340 | 1 | 1 | 5.29 |
| Ras-related protein Rab-6B | Q9NRW1 | 1 | 1 | 5.29 |
| Ras-related protein Rab-39A | Q14964 | 1 | 1 | 5.07 |
| Ras-related protein Rab-4A | P20338 | 1 | 1 | 5.05 |
| SRA stem-loop-interacting RNA-binding protein, mitochondrial | Q9GZT3 | 1 | 1 | 12.84 |
| Sterol-4-alpha-carboxylate 3-dehydrogenase, decarboxylating | Q15738 | 2 | 2 | 8.85 |
| Gamma-glutamyltransferase 5 | P36269 | 1 | 1 | 2.05 |
| Sequestosome-1 | Q13501 | 1 | 1 | 3.64 |
| Isoleucine--tRNA ligase, mitochondrial | Q9NSE4 | 1 | 1 | 1.48 |
| Macrophage migration inhibitory factor | P14174 | 1 | 1 | 7.83 |
| Nodal modulator 1 | Q15155 | 2 | 2 | 3.85 |
| Nodal modulator 2 | Q5JPE7 | 2 | 2 | 3.71 |
| Nodal modulator 3 | P69849 | 2 | 2 | 3.85 |
| Mitochondrial carrier homolog 2 | Q9Y6C9 | 1 | 1 | 4.29 |
| Transmembrane 9 superfamily member 3 | Q9HD45 | 1 | 1 | 4.41 |
| Rho-related GTP-binding protein RhoB | P62745 | 1 | 1 | 4.59 |
| Myosin-10 | P35580 | 1 | 1 | 0.96 |
| UDP-glucose:glycoprotein glucosyltransferase 1 | Q9NYU2 | 1 | 1 | 0.9 |
| Integral membrane protein 2B | Q9Y287 | 1 | 1 | 3.76 |
| Reticulon-3 | O95197 | 1 | 1 | 1.07 |
| Putative heat shock protein HSP 90-alpha A2 | Q14568 | 1 | 1 | 3.5 |
| Putative heat shock protein HSP 90-beta 2 | Q58FF8 | 1 | 1 | 3.15 |
| Cytochrome b-c1 complex subunit 10 | O14957 | 1 | 1 | 21.43 |
| Synaptophysin-like protein 1 | Q16563 | 1 | 1 | 4.25 |
| ER membrane protein complex subunit 2 | Q15006 | 1 | 1 | 4.04 |
| Pyruvate kinase PKLR | P30613 | 1 | 1 | 1.92 |
| Calumenin | O43852 | 1 | 1 | 4.13 |
| Cytochrome b-c1 complex subunit 8 | O14949 | 1 | 1 | 10.98 |
| Keratin, type I cytoskeletal 26 | Q7Z3Y9 | 1 | 1 | 1.92 |
| Delta-1-pyrroline-5-carboxylate synthase | P54886 | 1 | 1 | 1.51 |
| Myristoylated alanine-rich C-kinase substrate | P29966 | 1 | 1 | 5.72 |
| Sorting and assembly machinery component 50 homolog | Q9Y512 | 1 | 1 | 2.56 |
| 40S ribosomal protein S24 | P62847 | 1 | 1 | 9.02 |
| Keratin, type I cytoskeletal 18 | P05783 | 1 | 1 | 1.63 |
| Keratin, type I cuticular Ha7 | O76014 | 1 | 1 | 1.56 |
| Keratin, type I cuticular Ha3-II | Q14525 | 1 | 1 | 1.73 |
| Keratin, type I cuticular Ha6 | O76013 | 1 | 1 | 1.5 |
| Keratin, type I cytoskeletal 24 | Q2M2I5 | 1 | 1 | 1.33 |
| Keratin, type I cuticular Ha8 | O76015 | 1 | 1 | 1.54 |
| Keratin, type I cuticular Ha1 | Q15323 | 1 | 1 | 1.68 |
| Keratin, type I cuticular Ha5 | Q92764 | 1 | 1 | 1.54 |
| Keratin, type I cuticular Ha2 | Q14532 | 1 | 1 | 1.56 |
| Keratin, type I cytoskeletal 13 | P13646 | 1 | 1 | 1.53 |
| Annexin A11 | P50995 | 1 | 1 | 2.18 |
| NADH dehydrogenase [ubiquinone] iron-sulfur protein 6, mitochondrial | O75380 | 1 | 1 | 12.1 |
| Oligosaccharyltransferase complex subunit OSTC | Q9NRP0 | 1 | 1 | 8.05 |
| 60S ribosomal protein L13a | P40429 | 1 | 1 | 5.91 |
| AP-1 complex subunit beta-1 | Q10567 | 1 | 1 | 1.16 |
| AP-2 complex subunit beta | P63010 | 1 | 1 | 1.17 |
| Cytochrome c oxidase subunit 4 isoform 1, mitochondrial | P13073 | 1 | 2 | 6.51 |
| Microsomal glutathione S-transferase 3 | O14880 | 1 | 1 | 15.79 |
| Lysophospholipid acyltransferase 7 | Q96N66 | 1 | 1 | 3.6 |
| Serine palmitoyltransferase 2 | O15270 | 1 | 1 | 3.56 |
| Estradiol 17-beta-dehydrogenase 12 | Q53GQ0 | 1 | 1 | 4.81 |
| Ras-related C3 botulinum toxin substrate 2 | P15153 | 1 | 1 | 5.21 |
| Integrin alpha-1 | P56199 | 1 | 1 | 1.02 |
| HLA class I histocompatibility antigen, alpha chain G | P17693 | 1 | 1 | 3.85 |
| Mitochondrial import receptor subunit TOM40 homolog | O96008 | 1 | 1 | 9.42 |
| CD81 antigen | P60033 | 1 | 1 | 9.75 |
| Cytochrome c oxidase subunit 2 | P00403 | 1 | 1 | 8.81 |
| Endoplasmic reticulum-Golgi intermediate compartment protein 1 | Q969X5 | 1 | 1 | 8.28 |
| Splicing factor 3B subunit 3 | Q15393 | 1 | 1 | 0.99 |
| Insulin | P01308 | 1 | 1 | 19.09 |
| Sodium-coupled neutral amino acid transporter 2 | Q96QD8 | 1 | 1 | 4.15 |
| ERO1-like protein alpha | Q96HE7 | 1 | 1 | 2.99 |
| NAD(P)H dehydrogenase [quinone] 1 | P15559 | 1 | 1 | 3.65 |
| ADP-ribosylation factor-like protein 8A | Q96BM9 | 1 | 1 | 9.14 |
| ADP-ribosylation factor-like protein 8B | Q9NVJ2 | 1 | 1 | 9.14 |
| Chloride intracellular channel protein 4 | Q9Y696 | 1 | 1 | 4.74 |
| Phosphoglycerate kinase 1 | P00558 | 1 | 1 | 3.12 |
| Fatty aldehyde dehydrogenase | P51648 | 1 | 1 | 3.51 |
| Ras-related protein Rab-13 | P51153 | 1 | 1 | 5.42 |
| Guanine nucleotide-binding protein G(I)/G(S)/G(O) subunit gamma-5 | P63218 | 1 | 1 | 16.18 |
| Ubiquitin-60S ribosomal protein L40 | P62987 | 1 | 1 | 12.5 |
| Ubiquitin-40S ribosomal protein S27a | P62979 | 1 | 1 | 10.26 |
| Polyubiquitin-C | P0CG48 | 1 | 1 | 21.02 |
| Polyubiquitin-B | P0CG47 | 1 | 1 | 20.96 |
| Caveolin-1 | Q03135 | 1 | 1 | 4.49 |
| Serine palmitoyltransferase 1 | O15269 | 1 | 1 | 7.19 |
| ATP-dependent RNA helicase DDX39A | O00148 | 1 | 1 | 2.81 |
| Acyl-coenzyme A thioesterase 9, mitochondrial | Q9Y305 | 1 | 1 | 2.28 |
| Carnitine O-palmitoyltransferase 1, liver isoform | P50416 | 1 | 1 | 1.81 |
| Keratin, type II cytoskeletal 3 | P12035 | 1 | 1 | 1.43 |
| Monoacylglycerol lipase ABHD12 | Q8N2K0 | 1 | 1 | 4.77 |
| Tropomyosin alpha-1 chain | P09493 | 1 | 1 | 4.93 |
| Tropomyosin alpha-3 chain | P06753 | 1 | 1 | 4.91 |
| Tropomyosin alpha-4 chain | P67936 | 1 | 1 | 5.65 |
| Tropomyosin beta chain | P07951 | 1 | 1 | 4.93 |
| Dolichyl-diphosphooligosaccharide--protein glycosyltransferase subunit STT3B | Q8TCJ2 | 1 | 1 | 1.57 |
| 40S ribosomal protein S15a | P62244 | 1 | 1 | 10.77 |
| Peroxiredoxin-4 | Q13162 | 1 | 1 | 4.43 |
| B-cell receptor-associated protein 29 | Q9UHQ4 | 1 | 1 | 6.22 |
| Histone H3.1t | Q16695 | 1 | 1 | 5.15 |
| Histone H3.3 | P84243 | 1 | 1 | 5.15 |
| Histone H3.3C | Q6NXT2 | 1 | 1 | 5.19 |
| ADP-ribosylation factor-like protein 6-interacting protein 1 | Q15041 | 1 | 1 | 4.93 |
| Cysteine-rich with EGF-like domain protein 1 | Q96HD1 | 1 | 1 | 3.1 |
| Synaptic vesicle membrane protein VAT-1 homolog | Q99536 | 1 | 1 | 2.54 |
| Putative heat shock protein HSP 90-beta-3 | Q58FF7 | 1 | 1 | 1.84 |
| Histone H2B type 1-A | Q96A08 | 1 | 1 | 8.66 |
| C-1-tetrahydrofolate synthase, cytoplasmic | P11586 | 1 | 1 | 2.57 |
| 40S ribosomal protein S4, X isoform | P62701 | 1 | 1 | 3.8 |
| Protein disulfide-isomerase TMX3 | Q96JJ7 | 1 | 1 | 2.42 |
| GPI transamidase component PIG-T | Q969N2 | 1 | 1 | 2.25 |
| Dehydrogenase/reductase SDR family member 7 | Q9Y394 | 1 | 1 | 6.78 |
| Inositol monophosphatase 3 | Q9NX62 | 1 | 1 | 7.24 |
| Poly(rC)-binding protein 1 | Q15365 | 1 | 1 | 3.09 |
| Poly(rC)-binding protein 2 | Q15366 | 1 | 1 | 3.01 |
| Poly(rC)-binding protein 3 | P57721 | 1 | 1 | 2.96 |
| Nucleoside diphosphate kinase A | P15531 | 1 | 1 | 7.89 |
| Importin subunit beta-1 | Q14974 | 1 | 1 | 1.83 |
| NADH dehydrogenase [ubiquinone] 1 beta subcomplex subunit 10 | O96000 | 1 | 1 | 6.4 |
| Cell cycle control protein 50A | Q9NV96 | 1 | 1 | 3.6 |
| Talin-1 | Q9Y490 | 1 | 1 | 0.83 |
| Receptor expression-enhancing protein 5 | Q00765 | 1 | 1 | 4.23 |
| Beta/gamma crystallin domain-containing protein 3 | Q68DQ2 | 1 | 1 | 0.78 |
| DNA polymerase delta catalytic subunit | P28340 | 1 | 1 | 0.63 |
| Zinc finger protein 248 | Q8NDW4 | 1 | 1 | 1.55 |
| Thiamin pyrophosphokinase 1 | Q9H3S4 | 1 | 1 | 5.35 |
| ATP-dependent RNA helicase A | Q08211 | 1 | 1 | 1.26 |
| Maestro heat-like repeat-containing protein family member 6 | A6NGR9 | 1 | 1 | 0.83 |
| Guanine nucleotide-binding protein G(I)/G(S)/G(T) subunit beta-3 | P16520 | 1 | 1 | 2.06 |
| Glycine--tRNA ligase | P41250 | 1 | 1 | 1.35 |
| SEC14-like protein 2 | O76054 | 1 | 1 | 1.24 |
| Echinoderm microtubule-associated protein-like 6 | Q6ZMW3 | 1 | 1 | 0.26 |
| Echinoderm microtubule-associated protein-like 5 | Q05BV3 | 1 | 1 | 0.25 |
