# Supplementary material for: Human metapneumovirus Induces Reorganization of the Actin Cytoskeleton for Direct Cell-to-Cell Spread
Source: PLoS Pathog. 2016 Sep 28;12(9):e1005922. doi: 10.1371/journal.ppat.1005922 (PMC5040343; doi:10.1371/journal.ppat.1005922)
Supplement: S2 Table — (DOCX) [file ppat.1005922.s002.docx]

**Table S2. Proteomic identification of cellular proteins in supernatants from mock infected cells.**

| **Protein** | **Accession #** | **# of peptides** | **# of MS/MS Spectra** | **Coverage (%)** |
| --- | --- | --- | --- | --- |
| Keratin, type II cytoskeletal 1 | P04264 | 46 | 86 | 63.04 |
| Keratin, type I cytoskeletal 9 | P35527 | 42 | 75 | 72.55 |
| Keratin, type I cytoskeletal 10 | P13645 | 38 | 56 | 60.27 |
| Keratin, type II cytoskeletal 2 epidermal | P35908 | 31 | 37 | 63.69 |
| Keratin, type II cytoskeletal 6A | P02538 | 30 | 36 | 53.55 |
| Keratin, type II cytoskeletal 6C | P48668 | 29 | 35 | 51.77 |
| Keratin, type II cytoskeletal 6B | P04259 | 28 | 35 | 51.77 |
| Keratin, type I cytoskeletal 14 | P02533 | 30 | 36 | 66.1 |
| Keratin, type I cytoskeletal 16 | P08779 | 26 | 32 | 57.29 |
| Keratin, type II cytoskeletal 5 | P13647 | 26 | 32 | 46.1 |
| Keratin, type I cytoskeletal 17 | Q04695 | 23 | 26 | 45.6 |
| Hornerin | Q86YZ3 | 8 | 13 | 16.07 |
| Keratin, type II cytoskeletal 75 | O95678 | 10 | 11 | 13.07 |
| Desmoplakin | P15924 | 8 | 8 | 4.6 |
| Keratin, type II cytoskeletal 2 oral | Q01546 | 8 | 12 | 13.01 |
| Keratin, type I cytoskeletal 15 | P19012 | 8 | 8 | 10.75 |
| Protein S100-A9 | P06702 | 7 | 8 | 64.04 |
| Keratin, type II cytoskeletal 3 | P12035 | 5 | 7 | 9.87 |
| Keratin, type I cytoskeletal 13 | P13646 | 6 | 6 | 9.17 |
| Protein S100-A7 | P31151 | 4 | 4 | 35.64 |
| Keratin, type II cytoskeletal 8 | P05787 | 4 | 6 | 7.25 |
| Keratin, type I cytoskeletal 19 | P08727 | 6 | 6 | 8.75 |
| Keratin, type II cytoskeletal 1b | Q7Z794 | 3 | 5 | 5.54 |
| Keratin, type I cytoskeletal 28 | Q7Z3Y7 | 4 | 5 | 5.6 |
| Keratin, type II cytoskeletal 72 | Q14CN4 | 2 | 4 | 4.7 |
| Protein S100-A8 | P05109 | 4 | 4 | 37.63 |
| Keratin, type II cytoskeletal 79 | Q5XKE5 | 4 | 5 | 6.36 |
| Keratin, type II cytoskeletal 4 | P19013 | 3 | 3 | 6.74 |
| Calmodulin-like protein 5 | Q9NZT1 | 2 | 2 | 32.19 |
| Keratin, type II cytoskeletal 7 | P08729 | 3 | 4 | 5.33 |
| Keratin, type I cytoskeletal 25 | Q7Z3Z0 | 2 | 3 | 4 |
| Keratin, type I cytoskeletal 27 | Q7Z3Y8 | 2 | 3 | 3.92 |
| Annexin A2 | P07355 | 3 | 4 | 13.86 |
| Glyceraldehyde-3-phosphate dehydrogenase | P04406 | 3 | 3 | 16.72 |
| Keratin, type I cytoskeletal 20 | P35900 | 3 | 4 | 2.83 |
| Desmoglein-1 | Q02413 | 3 | 3 | 4.67 |
| Keratin, type II cytoskeletal 73 | Q86Y46 | 3 | 4 | 3.33 |
| Keratin, type II cytoskeletal 74 | Q7RTS7 | 3 | 4 | 3.4 |
| Protein S100-A7A | Q86SG5 | 2 | 2 | 23.76 |
| Junction plakoglobin | P14923 | 2 | 2 | 3.89 |
| Fatty acid-binding protein, epidermal | Q01469 | 2 | 2 | 25.93 |
| Keratin, type I cytoskeletal 24 | Q2M2I5 | 2 | 2 | 3.43 |
| Keratin, type II cuticular Hb4 | Q9NSB2 | 2 | 3 | 2.33 |
| Putative annexin A2-like protein | A6NMY6 | 2 | 3 | 9.73 |
| Actin, cytoplasmic 1 | P60709 | 2 | 2 | 7.73 |
| Actin, cytoplasmic 2 | P63261 | 2 | 2 | 7.73 |
| Keratin, type I cytoskeletal 12 | Q99456 | 2 | 2 | 1.82 |
| Glial fibrillary acidic protein | P14136 | 1 | 1 | 2.55 |
| Keratin, type II cytoskeletal 80 | Q6KB66 | 1 | 1 | 2.43 |
| Keratinocyte proline-rich protein | Q5T749 | 2 | 2 | 5.7 |
| Neurofilament heavy polypeptide | P12036 | 2 | 2 | 0.88 |
| Keratin, type I cytoskeletal 26 | Q7Z3Y9 | 1 | 1 | 1.92 |
| Keratin, type II cytoskeletal 71 | Q3SY84 | 1 | 2 | 1.72 |
| Filaggrin-2 | Q5D862 | 1 | 1 | 0.46 |
| Keratin, type II cytoskeletal 78 | Q8N1N4 | 2 | 2 | 3.85 |
| Keratin, type I cytoskeletal 23 | Q9C075 | 1 | 1 | 2.13 |
| Keratin-like protein KRT222 | Q8N1A0 | 1 | 1 | 3.05 |
| Cathepsin D | P07339 | 1 | 1 | 2.67 |
| Protein S100-A11 | P31949 | 1 | 1 | 15.24 |
| Actin, alpha skeletal muscle | P68133 | 1 | 1 | 2.92 |
| Actin, gamma-enteric smooth muscle | P63267 | 1 | 1 | 2.93 |
| Actin, alpha cardiac muscle 1 | P68032 | 1 | 1 | 2.92 |
| Actin, aortic smooth muscle | P62736 | 1 | 1 | 2.92 |
| POTE ankyrin domain family member I | P0CG38 | 1 | 1 | 1.02 |
| POTE ankyrin domain family member F | A5A3E0 | 1 | 1 | 1.02 |
| POTE ankyrin domain family member E | Q6S8J3 | 1 | 1 | 1.02 |
| Alpha-enolase | P06733 | 1 | 1 | 4.15 |
| Beta-enolase | P13929 | 1 | 1 | 4.15 |
| Gamma-enolase | P09104 | 1 | 1 | 4.15 |
| Arginase-1 | P05089 | 1 | 1 | 6.52 |
| Microtubule-associated protein 1B | P46821 | 1 | 2 | 0.41 |
| Keratin, type I cytoskeletal 18 OS=Homo sapiens GN=KRT18 PE=1 SV=2 - [K1C18_HUMAN] | P05783 | 1 | 1 | 1.63 |
| Keratin, type I cuticular Ha7 | O76014 | 1 | 1 | 1.56 |
| Keratin, type I cuticular Ha3-II | Q14525 | 1 | 1 | 1.73 |
| Keratin, type I cuticular Ha6 | O76013 | 1 | 1 | 1.5 |
| Keratin, type I cuticular Ha8 | O76015 | 1 | 1 | 1.54 |
| Keratin, type I cuticular Ha1 | Q15323 | 1 | 1 | 1.68 |
| Keratin, type I cuticular Ha5 | Q92764 | 1 | 1 | 1.54 |
| Keratin, type I cuticular Ha2 | Q14532 | 1 | 1 | 1.56 |
| Peroxiredoxin-1 | Q06830 | 1 | 1 | 5.53 |
| Peroxiredoxin-2 | P32119 | 1 | 1 | 5.56 |
| Calmodulin-regulated spectrin-associated protein 2 | Q08AD1 | 1 | 1 | 0.67 |
| Arf-GAP with SH3 domain, ANK repeat and PH domain-containing protein 2 | O43150 | 1 | 1 | 0.8 |
| Transmembrane and coiled-coil domain-containing protein 6 | Q96DC7 | 1 | 1 | 3.04 |
| Transcription factor 15 | Q12870 | 1 | 1 | 5.03 |
| DnaJ homolog subfamily C member 15 | Q9Y5T4 | 1 | 1 | 6 |
| Plasminogen activator inhibitor 2 | P05120 | 1 | 1 | 2.89 |
| Cyclin-dependent kinase 7 | P50613 | 1 | 1 | 2.6 |
| Bifunctional ATP-dependent dihydroxyacetone kinase/FAD-AMP lyase (cyclizing) | Q3LXA3 | 1 | 1 | 1.91 |
| Zinc finger protein 878 | C9JN71 | 1 | 1 | 1.69 |
| Amiloride-sensitive sodium channel subunit delta | P51172 | 1 | 1 | 1.41 |
| Serpin B3 | P29508 | 1 | 1 | 6.41 |
| Serpin B4 | P48594 | 1 | 1 | 6.41 |
